# Preoperative prediction of diffuse glioma type and grade in adults: a gadolinium-free MRI-based decision tree

**DOI:** 10.1007/s00330-024-11140-5

**Published:** 2024-10-19

**Authors:** Aynur Azizova, Yeva Prysiazhniuk, Ivar J. H. G. Wamelink, Marcus Cakmak, Elif Kaya, Pieter Wesseling, Philip C. de Witt Hamer, Niels Verburg, Jan Petr, Frederik Barkhof, Vera C. Keil

**Affiliations:** 1 Amsterdam UMC Location Vrije Universiteit Amsterdam, Radiology & Nuclear Medicine Department, Amsterdam, The Netherlands; 2 Cancer Center Amsterdam, Imaging and Biomarkers, Amsterdam, The Netherlands; 3 Charles University, The Second Faculty of Medicine, Department of Pathophysiology, Prague, Czech Republic; 4 Motol University Hospital, Prague, Czech Republic; 5 Vrije Universiteit Amsterdam, University Medical Center, Amsterdam, The Netherlands; 6 Ankara Yıldırım Beyazıt University, Faculty of Medicine, Ankara, Turkey; 7 Amsterdam UMC location Vrije Universiteit Amsterdam, Department of Pathology, Amsterdam, The Netherlands; 8 Princess Máxima Center for Pediatric Oncology, Laboratory for Childhood Cancer Pathology, Utrecht, The Netherlands; 9 Amsterdam UMC location Vrije Universiteit Amsterdam, Department of Neurosurgery, Brain Tumor Center Amsterdam, Amsterdam, The Netherlands; 10 Helmholtz-Zentrum Dresden-Rossendorf, Institute of Radiopharmaceutical Cancer Research, Dresden, Germany; 11 Amsterdam Neuroscience, Brain Imaging, Amsterdam, The Netherlands; 12 Queen Square Institute of Neurology and Center for Medical Image Computing, University College London, London, UK

**Keywords:** Brain neoplasms, Glioma, Isocitrate dehydrogenase, Gadolinium, Magnetic resonance imaging

## Abstract

**Objectives:**

To develop a gadolinium-free MRI-based diagnosis prediction decision tree (DPDT) for adult-type diffuse gliomas and to assess the added value of gadolinium-based contrast agent (GBCA) enhanced images.

**Materials and methods:**

This study included preoperative grade 2–4 adult-type diffuse gliomas (World Health Organization 2021) scanned between 2010 and 2021. The DPDT, incorporating eleven GBCA-free MRI features, was developed using 18% of the dataset based on consensus readings. Diagnosis predictions involved grade (grade 2 vs. grade 3/4) and molecular status (isocitrate dehydrogenase (IDH) and 1p/19q). GBCA-free diagnosis was predicted using DPDT, while GBCA-enhanced diagnosis included post-contrast images. The accuracy of these predictions was assessed by three raters with varying experience levels in neuroradiology using the test dataset. Agreement analyses were applied to evaluate the prediction performance/reproducibility.

**Results:**

The test dataset included 303 patients (age (SD): 56.7 (14.2) years, female/male: 114/189, low-grade/high-grade: 54/249, IDH-mutant/wildtype: 82/221, 1p/19q-codeleted/intact: 34/269). Per-rater GBCA-free predictions achieved ≥ 0.85 (95%-CI: 0.80–0.88) accuracy for grade and ≥ 0.75 (95%-CI: 0.70–0.80) for molecular status, while GBCA-enhanced predictions reached ≥ 0.87 (95%-CI: 0.82–0.90) and ≥ 0.77 (95%–CI: 0.71–0.81), respectively. No accuracy difference was observed between GBCA-free and GBCA-enhanced predictions. Group inter-rater agreement was moderate for GBCA-free (0.56 (95%-CI: 0.46–0.66)) and substantial for GBCA-enhanced grade prediction (0.68 (95%-CI: 0.58–0.78), *p* = 0.008), while substantial for both GBCA-free (0.75 (95%-CI: 0.69–0.80) and GBCA-enhanced (0.77 (95%-CI: 0.71–0.82), *p* = 0.51) molecular status predictions.

**Conclusion:**

The proposed GBCA-free diagnosis prediction decision tree performed well, with GBCA-enhanced images adding little to the preoperative diagnostic accuracy of adult-type diffuse gliomas.

**Key Points:**

***Question*** *Given health and environmental concerns, is there a gadolinium-free imaging protocol to preoperatively evaluate gliomas comparable to the gadolinium-enhanced standard practice?*

***Findings*** *The proposed gadolinium-free diagnosis prediction decision tree for adult-type diffuse gliomas performed well, and gadolinium-enhanced MRI demonstrated only limited improvement in diagnostic accuracy.*

***Clinical relevance*** *Even inexperienced raters effectively classified adult-type diffuse gliomas using the gadolinium-free diagnosis prediction decision tree, which, until further validation, can be used alongside gadolinium-enhanced images to respect standard practice, despite this study showing that gadolinium-enhanced images hardly improved diagnostic accuracy*.

**Graphical Abstract:**

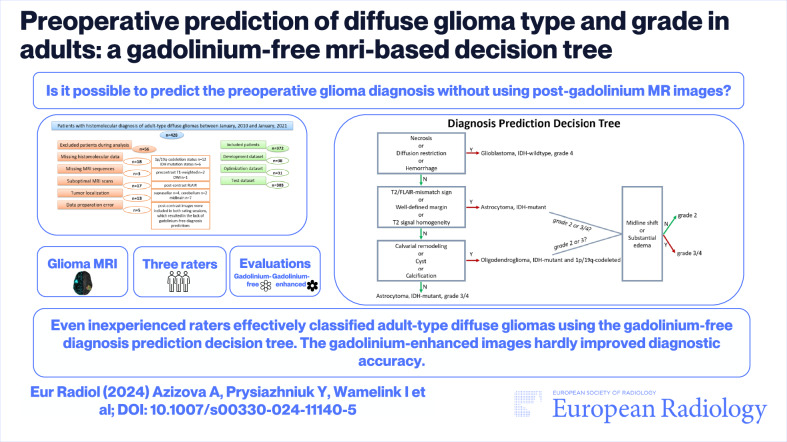

## Introduction

Gadolinium-based contrast agent (GBCA)-enhanced MRI is the current standard imaging modality for managing brain tumors, including adult-type diffuse gliomas, aiding diagnosis and treatment decisions [1]. Nonetheless, enhancement is an imperfect measure for both tumor malignancy and resectability of tumor borders [2]. Tumors displaying enhancement may not always be high-grade gliomas [3]; conversely, high-grade gliomas may lack enhancement [4]. This conflict is acknowledged in the latest Response Assessment in Neuro-oncology criteria (RANO 2.0), which also stresses the diagnostic relevance of GBCA-free sequences [5].

While being a standard imaging practice [1], GBCA increasingly raises concerns about associated side effects, with safety recommendations relying solely on expert opinion rather than prospective experimental evidence [6]. Although certain linear GBCAs were restricted due to their link with nephrogenic systemic fibrosis, renal impairment remains the primary catalyst for this condition, with uncertainty about whether normal renal function excludes the risk [6]. With uncertain clinical implications, GBCA, mainly in linear forms, was also identified to accumulate in the body [7]. Furthermore, studies indicate anthropogenic medical gadolinium accumulation in ecosystems, raising concerns about aquatic life and urban water safety [8]. Beyond these challenges, longer examination times, increased financial costs [9], and limited availability in low-middle-income countries [10] are stimuli to the shift from GBCA-enhanced MRI to GBCA-free MRI. Additionally, vulnerable populations, such as pregnant or breastfeeding women [11] and children [12], necessitate careful consideration due to putative GBCA exposure risks.

Various artificial intelligence (AI) methods hold the potential for substituting GBCA with synthetic GBCA-enhanced images [13] or reducing contrast dosage through augmented GBCA-enhanced images [14], but integration into clinical practice is lagging. While advanced imaging techniques like arterial spin labeling (ASL) [15] or amide proton transfer chemical exchange saturation transfer (APT-CEST) [16] introduce alternative GBCA-free parameters, their utilization is constrained by availability and variability in acquisition parameters. Conversely, conventional MRI sequences are a component of daily practice and provide essential glioma imaging biomarkers, such as T2-FLAIR mismatch signs or cysts, many of which can be assessed without GBCA-enhanced images [17]. However, previous studies predominantly assessed these biomarkers, such as necrosis [18–20], with GBCA-enhanced MRI, as it has been the standard of care, leaving open questions about the predictive added value of GBCA-enhanced images. Further maturation of AI-based and advanced MRI methods and their clinical translation into glioma management will be a long process. Therefore, qualitative parameter evaluation of conventional GBCA-free MRI, combined with a simple decision tree, might be the near-future solution for phasing out GBCA use in glioma, as it is more time-efficient than quantitative approaches.

As a first step to develop and establish a general GBCA-free MRI-based diagnosis prediction decision tree (DPDT) for brain tumors, this study aims to assess the additive value of GBCA-enhanced images in predicting histomolecular diagnosis in adult-type diffuse gliomas.

## Materials and methods

### Study sample

This retrospective single-center study received approval from the institutional medical ethics review board (Vumc_2021-0437). Informed consent was waived. Eligible patient cases from the hospital glioma database (IMAGO) registered from January 2010 to January 2021 were consecutively added to the trial database. The eligibility criteria are listed in Table 1.

**Table 1:** Eligibility criteria

Inclusion criteria	Exclusion criteria
(a) patients with grade 2–4 adult-type diffuse gliomas based on the 5th WHO-CNS tumor classification (b) presence of IDH mutation and 1p/19q-codeletion status (c) no more than one month gap between preoperative MRI and surgery (d) availability of the following mandatory MRI sequences: pre-contrast T1-weighted, T2-weighted, FLAIR, DWI/ADC, and post-contrast T1-weighted	(a) pediatric patients (b) missing/incomplete histopathological diagnosis (c) suprasellar, midline, and cerebellar tumors as adult-type diffuse gliomas are rare and may have distinct radiological features in these locations (d) missing MRI sequences (e) MRI scans with suboptimal quality, including movement-related artifacts (f) patients who declined permission for their data to be used in research during their original stay at the institution (scientific use opt-out) (g) data preparation errors during the randomization of gadolinium-free and gadolinium-enhanced images of the same patients into separate evaluation sessions

Table 1 describes the inclusion and exclusion criteria for this study

*DWI/ADC* diffusion-weighted imaging/apparent diffusion coefficient, *FLAIR* fluid-attenuated inversion recovery, *IDH* isocitrate dehydrogenase, *WHO-CNS* World Health Organization-central nervous system

### MRI and datasets

Seven MRI scanners provided the images used in this study (Table S1). I.W., a fourth-year Ph.D. candidate in neuro-oncology, conducted data preparation, including pseudonymization. Eligible patients were randomly assigned into three subsets: development (*n* = 38), optimization (*n* = 31), and test (*n* = 303). RADIANT software facilitated access to pseudonymized datasets (3.4.1.13367; https://www.radiantviewer.com/). Two raters (V.K., 11 years of neuroradiology experience; A.A., 5 years of neuroradiology experience) explored development and optimization datasets. The test dataset was independently assessed, blinded to the reference standard, by three raters (V.K., A.A., and M.C.). Rater 3 (M.C.) was a fourth-year medical student without prior radiology experience who underwent training using the optimization dataset.

#### Reference standard

Histomolecular diagnosis, based on the 2021 World Health Organization classification, served as the reference standard. Isocitrate dehydrogenase (IDH) status was determined via immunohistochemistry, next-generation sequencing, and/or methylation profiling, and 1p/19q-codeletion status was assessed using loss of heterozygosity (LOH) analysis or methylation profiling. The final histomolecular diagnosis of glioblastoma in IDH-wildtype cases was determined based on additional molecular markers (e.g., TERT promoter mutation, EGFR amplification, and combination of chromosome 7 gain and chromosome 10 loss) and supporting histological features (e.g., necrosis, microvascular proliferation, and high mitotic index). A small subset of IDH-wildtype diffuse gliomas (*n* = 16) that lacked molecular analysis (not otherwise specified) or had negative molecular markers (not elsewhere classified) were included in the study as glioblastoma, IDH-wildtype based on their final multidisciplinary team diagnosis indicating aggressive clinical behavior. Grade 2 gliomas were categorized as low-grade (LGG), while grade 3/4 as high-grade (HGG). IDH-wildtype diffuse gliomas were accepted as HGG regardless of their histological grade because of their generally aggressive clinical behavior.

### Diagnosis prediction decision tree (DPDT)

**Fig. 1:**
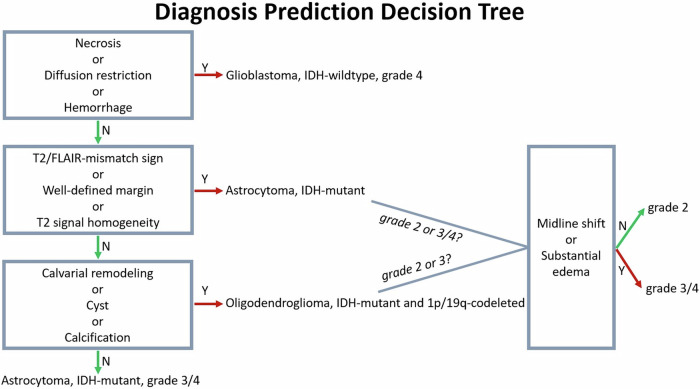
Diagnosis prediction decision tree (DPDT) based on GBCA-free MRI sequences. Flow chart describes DPDT for adult-type diffuse gliomas encompassing seven VASARI (necrosis, diffusion, hemorrhage, non-enhancing tumor margin, calvarial remodeling, cysts, proportion of edema) and four non-VASARI (T2-FLAIR mismatch sign, T2 signal homogeneity, calcification, midline shift) imaging features guiding the histomolecular diagnosis decision. GBCA, gadolinium-based contrast agent; VASARI, Visually AcceSAble Rembrandt Images

Two raters assessed a development dataset initially comprising only GBCA-free MRI scans (pre-contrast T1-weighted, T2-weighted, FLAIR, DWI/ADC, and SWI (if present)) to predict histomolecular diagnoses: (1) glioma grade (LGG vs. HGG) and (2) molecular status (astrocytoma, IDH-mutant vs. oligodendroglioma, IDH-mutant and 1p/19q-codeleted vs. glioblastoma, IDH-wildtype). One week later, raters reexamined all cases, integrating the post-contrast T1-weighted sequence to evaluate the added value of the GBCA-enhanced scans using common clinical radiology practice. For instance, tumors with avid enhancement or rim enhancement were assigned to HGG and glioblastoma, IDH-wildtype groups, respectively. In both rating rounds, they justified their decisions on a case-by-case basis by identifying key imaging features, drawing from individual clinical experience, and utilizing literature-based biomarkers [18–25], as well as the Visually AcceSAble Rembrandt Images (VASARI), glioma imaging features set [26]. Subsequently, the raters collaboratively analyzed the results to identify helpful biomarkers for GBCA-free diagnosis prediction correlated with the reference standard. Following this consensus, a DPDT comprising seven VASARI (necrosis, diffusion, hemorrhage, non-enhancing tumor margin, calvarial remodeling, cysts, proportion of edema) and four non-VASARI (T2-FLAIR mismatch sign, T2 signal homogeneity, calcification, midline shift) imaging features, each previously linked to the respective histomolecular diagnosis [18–25], was proposed; see Fig. 1, Table S2, and Supplementary material. Figs. S1–S5 depicts case examples for DPDT imaging features.

The optimization dataset was assessed, both with and without GBCA-enhanced images, at a one-week interval to gauge the effectiveness of DPDT using only the exclusive imaging features included in this tree, its impact on inter-rater agreement, and its potential applicability in a larger cohort. The GBCA-free diagnosis was evaluated using DPDT based on GBCA-free MRI sequences, while the GBCA-enhanced diagnosis included the post-contrast T1-weighted images alongside the DPDT.

### Test dataset

Three raters (V.K., A.A., M.C.) assessed the diagnoses using the GBCA-free *and* GBCA-enhanced DPDT in a larger cohort to compare the predictive diagnostic accuracy using GBCA-free vs. GBCA-enhanced scans. A DPDT guide including definitions of imaging features, was provided to raters (Supplementary material). MRI scans were randomly distributed across two rating sessions. The first session assessed GBCA-free and GBCA-enhanced scans from different patients. In the second session, scans from the same patients, which had not yet been rated as GBCA-enhanced or GBCA-free, were evaluated in a differently randomized order. This approach aimed to mitigate confirmation bias by ensuring that GBCA-free or GBCA-enhanced scans were not exclusively assessed in the same session.

### Statistical analysis

Rater prediction performance was evaluated using overall accuracy for multiple classes (astrocytoma, IDH-mutant vs. oligodendroglioma, IDH-mutant and 1p/19q-codeleted vs. glioblastoma, IDH-wildtype), along with accuracy, sensitivity, specificity, and negative and positive predictive values for binary classes (e.g., HGG vs. LGG or astrocytoma, IDH-mutant vs. others). The performance between GBCA-free and GBCA-enhanced datasets was compared using McNemar’s test with Yates continuity correction [27].

Prediction performance was also assessed across different subgroups, including age, sex, tumor location, and tumor laterality (right/left/midline), to identify factors that might influence diagnostic accuracy. Logistic regression and Pearson’s Chi-squared test were used for continuous and categorical variables, respectively. This subgroup analysis was conducted on a combined dataset of all raters, with separate evaluations for GBCA-free and GBCA-enhanced scans.

Inter-rater agreement was analyzed both collectively and pairwise among the raters. Kendall’s *W* and Fleiss’ kappa were used for group inter-rater agreement, and weighted and unweighted Cohen’s kappa were used for pairwise inter-rater/intra-rater inter-group agreements in ordered and unordered features, respectively. Intra-rater inter-group agreements examined the consistency within raters by comparing the GBCA-free and GBCA-enhanced predictions. Unweighted Cohen’s kappa was supplemented with prevalence-adjusted and bias-adjusted kappa (PABAK) for binary features and between two groups to compensate for a possible influence of dataset diagnosis imbalances due to naturally different tumor incidence rates [28].

The interpretation of agreement values was as follows: 0.01–0.20, slight; 0.21–0.40, fair; 0.41–0.60, moderate; 0.61–0.80, substantial; and 0.81–0.99, almost perfect [29]. The comparison of agreements was conducted using the *Z*-test or Hotelling’s T2 test [30] with the “multiagree” R package.

Data analysis was conducted by Y.P., a third-year Ph.D. student in neuroscience, using R package 4.3.0. Bootstrapping and PABAK calculations were performed using “multiagree” R and epiR (2.0.68 https://CRAN.R-project.org/package=epiR) packages, respectively. The significance threshold was *p* < 0.05.

## Results

**Fig. 2:**
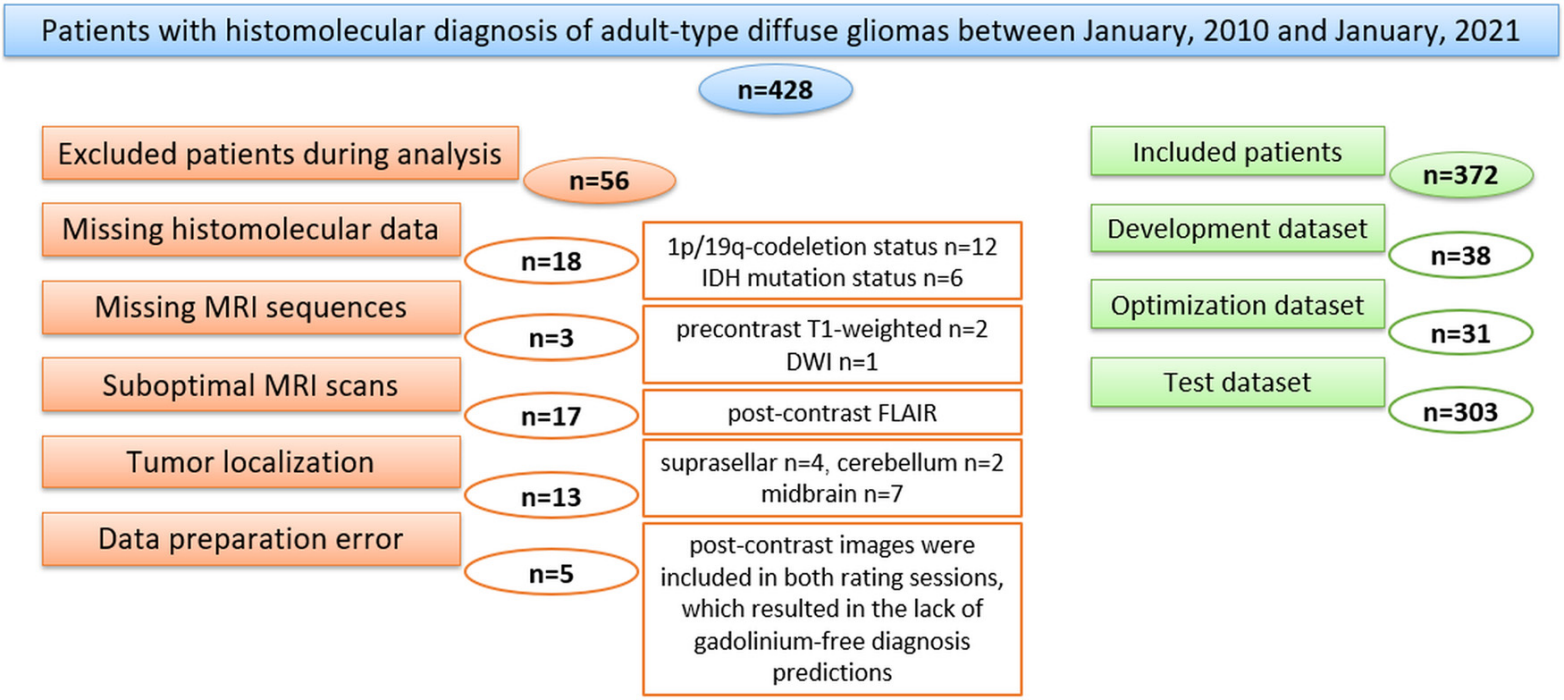
Patient enrollment diagram. The flow chart depicts the patients included and excluded in this study. IDH, isocitrate dehydrogenase

Figure 2 illustrates the patient cohort. Table 2 lists the demographics of the study cohort.

**Table 2:** Patient demographics

Demographics	Datasets		
	Development	Optimization	Test
Sample size	*n* = 38	*n* = 31	*n* = 303
Age (SD) (years)	59 (15.9)	52 (14.9)	56.7 (14.2)
Sex (female/male)	16/22	14/17	114/189
Histological grade (LGG/HGG)	5/33	9/22	54/249
IDH mutation status (IDH-mutant/IDH-wildtype)	6/32	16/15	82/221
1p/19q-codeletion status (codeleted/intact)	1/37	7/24	34/269

Table 2 describes the main demographics for the development, optimization, and test datasets

*IDH* isocitrate dehydrogenase, *HGG* high-grade glioma (grade 3/4), *LGG* low-grade glioma (grade 2), *SD* standard deviation

### Development and optimization datasets

The raters’ GBCA-free prediction performance improved in the optimization dataset compared to the development dataset. For instance, the overall accuracy of GBCA-free molecular diagnosis prediction for raters 1 and 2 increased from 0.76 and 0.74 in the development dataset to 0.81 and 0.84 in the optimization dataset, respectively (Tables S3/S4 and Fig. S6/7). The comparison of GBCA-free and GBCA-enhanced histomolecular predictions per rater revealed no significant differences in both development and optimization datasets (all *p* > 0.05).

Following the implementation of DPDT, there was an improvement in inter-rater agreement in GBCA-free molecular diagnosis prediction in the development (0.45 (95%-CI: 0.18–0.71)) and optimization (0.78 (95%-CI: 0.57–0.98)) datasets, showing a trend towards significance (*p* = 0.06). Slight improvements were observed in the GBCA-free grade prediction (0.80 (95%-CI: 0.54–1.06) vs. 0.85 (95%-CI: 0.64–1.05), *p* = 0.81), as well as GBCA-enhanced molecular diagnosis (0.51 (95%-CI: 0.23–0.79) vs. 0.64 (95%-CI: 0.42–0.87), *p* = 0.50) and GBCA-enhanced grade (0.61 (95%-CI: 0.28–0.94) vs. 0.74 (95%-CI: 0.46–1.01), *p* = 0.59) prediction.

### Test dataset

#### Predictive performance of the raters using GBCA-free vs. GBCA-enhanced scans

**Fig. 3:**
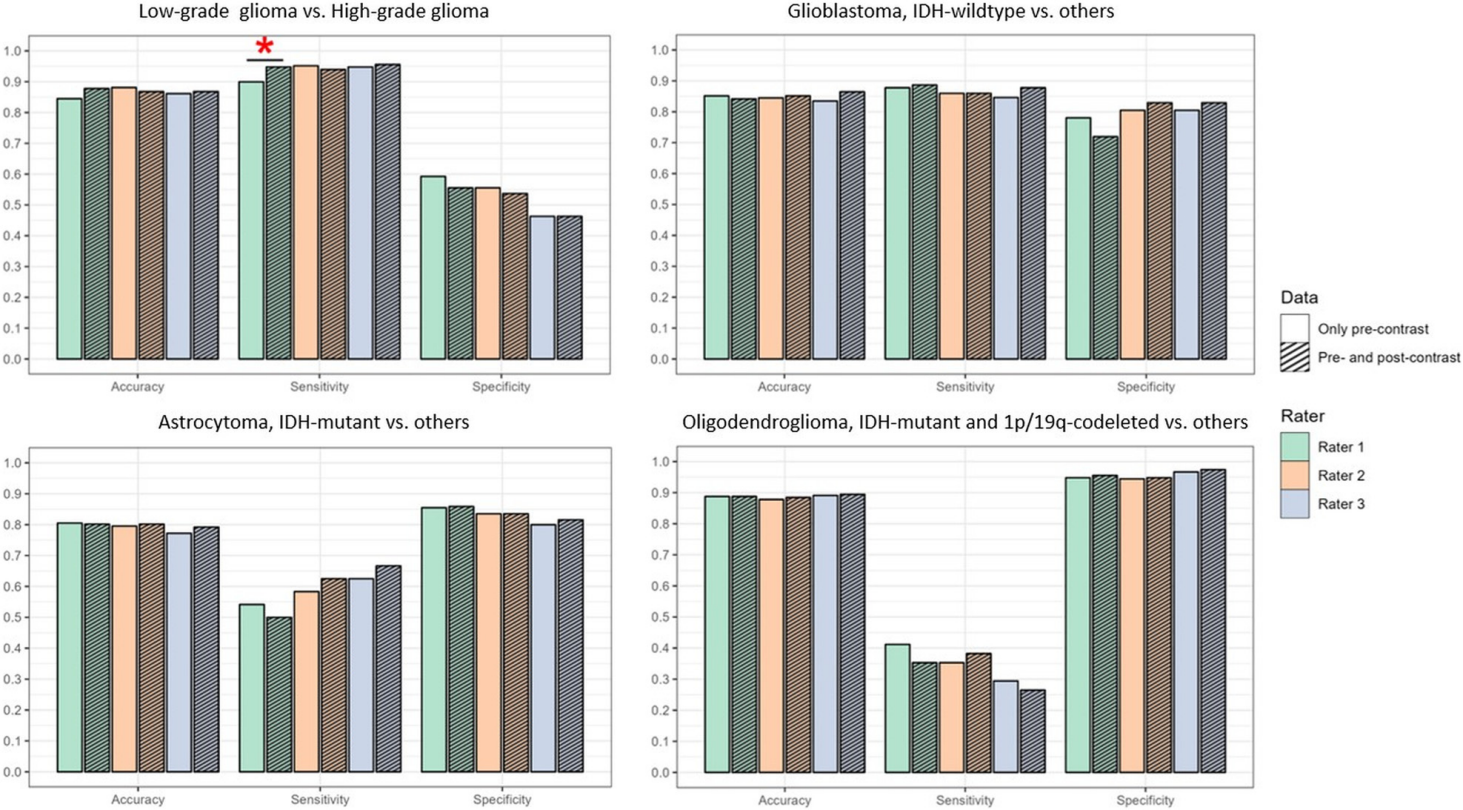
Per-rater prediction performance of histomolecular diagnosis of adult-type diffuse gliomas using GBCA-free vs. GBCA-enhanced scans. Color bar charts show the prediction performance, including accuracy, sensitivity, and specificity levels in predicting tumor grade, IDH mutation, and 1p/19q-codeletion status using GBCA-free and GBCA-enhanced scans per rater (rater 1 = green bar, rater 2 = orange bar, rater 3 = blue bar). Comparison of GBCA-free and GBCA-enhanced predictions revealed no significant differences (*p* > 0.05) except for rater 1’s sensitivity in histopathological grade prediction (red star, *p* = 0.006). GBCA, gadolinium-based contrast agent

The accuracy in predicting tumor grade (LGG vs. HGG) using GBCA-free scans was 0.85 (95%-CI: 0.80–0.88), 0.88 (95%-CI: 0.84–0.92), and 0.86 (95%-CI: 0.82–0.90) for raters 1, 2, and 3, respectively. The corresponding accuracies for GBCA-enhanced scans were 0.88 (95%-CI: 0.84–0.91), 0.87 (95%-CI: 0.82–0.90), and 0.87 (95%-CI: 0.83–0.90). Regarding using GBCA-free scans for predicting the molecular status, the overall accuracies were 0.77 (95%-CI: 0.72–0.82), 0.76 (95%-CI: 0.71–0.81), and 0.75 (95%-CI: 0.70–0.80) for raters 1, 2, and 3, respectively. The corresponding overall accuracies for GBCA-enhanced scans were 0.77 (95%-CI: 0.71–0.81), 0.77 (95%-CI: 0.72–0.82), and 0.78 (95%-CI: 0.72–0.82). Comparing the GBCA-free and GBCA-enhanced outcomes revealed insignificant differences (all *p* > 0.05) except for rater 1’s sensitivity in grade prediction (GBCA-free/GBCA-enhanced 0.90/0.95, *p* = 0.006); see Table 3, Fig. 3, and Fig. S8.

**Table 3:** Prediction performance of the raters in the test dataset

Results		Rater 1	Rater 2	Rater 3
Histopathological grade with GBCA-free MRI				
Accuracy		0.85	0.88	0.86
Sensitivity		0.90	0.95	0.95
Specificity		0.59	0.56	0.46
Positive predictive value		0.91	0.91	0.89
Negative predictive value		0.56	0.71	0.66
Histopathological grade with GBCA-enhanced MRI				
Accuracy		0.88	0.87	0.87
Sensitivity		0.95	0.94	0.96
Specificity		0.56	0.54	0.46
Positive predictive value		0.91	0.90	0.89
Negative predictive value		0.70	0.66	0.69
Molecular diagnosis with GBCA-free MRI				
Overall accuracy (astrocytoma, IDH-mutant vs. oligodendroglioma, IDH-mutant and 1p/19q-codeleted vs. glioblastoma, IDH-wildtype)		0.77	0.76	0.75
Accuracy	Astrocytoma, IDH-mutant	0.81	0.80	0.78
	Oligodendroglioma, IDH-mutant and 1p/19q-codeleted	0.89	0.88	0.89
	Glioblastoma, IDH-wildtype	0.85	0.85	0.83
Sensitivity	Astrocytoma, IDH-mutant	0.54	0.58	0.63
	Oligodendroglioma, IDH-mutant and 1p/19q-codeleted	0.42	0.35	0.29
	Glioblastoma, IDH-wildtype	0.88	0.86	0.85
Specificity	Astrocytoma, IDH-mutant	0.85	0.84	0.80
	Oligodendroglioma, IDH-mutant and 1p/19q-codeleted	0.95	0.94	0.97
	Glioblastoma, IDH-wildtype	0.78	0.80	0.80
Positive predictive value	Astrocytoma, IDH-mutant	0.41	0.40	0.37
	Oligodendroglioma, IDH-mutant and 1p/19q-codeleted	0.50	0.44	0.53
	Glioblastoma, IDH-wildtype	0.92	0.92	0.92
Negative predictive value	Astrocytoma, IDH-mutant	0.91	0.91	0.92
	Oligodendroglioma, IDH-mutant and 1p/19q-codeleted	0.93	0.92	0.92
	Glioblastoma, IDH-wildtype	0.70	0.68	0.61
Molecular diagnosis with GBCA-enhanced MRI				
Overall accuracy (astrocytoma, IDH-mutant vs. oligodendroglioma, IDH-mutant and 1p/19q-codeleted vs. glioblastoma, IDH-wildtype)		0.77	0.77	0.78
Accuracy	Astrocytoma, IDH-mutant	0.80	0.80	0.79
	Oligodendroglioma, IDH-mutant and 1p/19q-codeleted	0.89	0.88	0.89
	Glioblastoma, IDH-wildtype	0.84	0.85	0.86
Sensitivity	Astrocytoma, IDH-mutant	0.50	0.63	0.67
	Oligodendroglioma, IDH-mutant and 1p/19q-codeleted	0.35	0.38	0.26
	Glioblastoma, IDH-wildtype	0.89	0.86	0.88
Specificity	Astrocytoma, IDH-mutant	0.86	0.84	0.82
	Oligodendroglioma, IDH-mutant and 1p/19q-codeleted	0.96	0.95	0.97
	Glioblastoma, IDH-wildtype	0.72	0.83	0.83
Positive predictive value	Astrocytoma, IDH-mutant	0.40	0.42	0.41
	Oligodendroglioma, IDH-mutant and 1p/19q-codeleted	0.50	0.48	0.56
	Glioblastoma, IDH-wildtype	0.90	0.93	0.93
Negative predictive value	Astrocytoma, IDH-mutant	0.90	0.92	0.93
	Oligodendroglioma, IDH-mutant and 1p/19q-codeleted	0.92	0.92	0.91
	Glioblastoma, IDH-wildtype	0.70	0.69	0.72

Table 3 describes the diagnostic prediction performance, with and without GBCA-enhanced scans, per rater in the test dataset (*n* = 303)

Evaluations with GBCA-free MRI were based on pre-contrast T1-weighted, T2-weighted, FLAIR, DWI/ADC, and SWI (if present) sequences using the Diagnosis Prediction Decision Tree (DPDT). Evaluations with GBCA-enhanced MRI included post-contrast T1-weighted images in addition to the assessment conducted with GBCA-free MRI

*IDH* isocitrate dehydrogenase, *GBCA* gadolinium-based contrast agent

#### Predictive performance in different subgroups using GBCA-free and GBCA-enhanced scans

Subgroup analysis showed a significant correlation between the prediction of tumor grade or molecular status and patient age. The diagnostic accuracy improved with increasing patient age, regardless of whether GBCA-free or GBCA-enhanced scans were used (all *p* < 0.001). Similarly, a significant correlation was observed between GBCA-free or GBCA-enhanced diagnosis predictions and tumor location (all *p* < 0.001). Tumors in the thalamus were more frequently misclassified than those in other locations, particularly the parietal and temporal lobes; see Table S5. Predictions did not vary between patient sex or tumor laterality subgroups (all *p* > 0.05).

#### Agreement analysis for histomolecular diagnosis

**Fig. 4:**
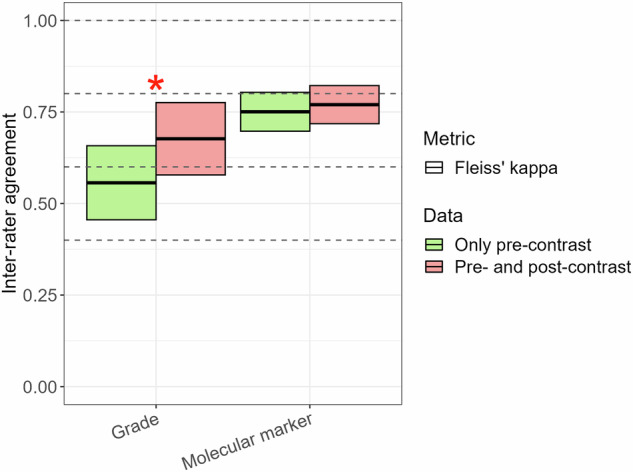
Group inter-rater agreement in histomolecular diagnosis prediction of adult-type diffuse gliomas using GBCA-free vs. GBCA-enhanced scans. The color box plot shows inter-rater agreement in predicting tumor grade (low-grade: grade 2 vs. high-grade: grade 3/4) and molecular status (astrocytoma, IDH-mutant vs. oligodendroglioma, IDH-mutant and 1p/19q-codeleted vs. glioblastoma, IDH-wildtype) among all raters. Green bars depict the results based on the evaluation of GBCA-free scans (only pre-contrast sequences: pre-contrast T1-weighted, T2-weighted, FLAIR, DWI/ADC, and SWI (if present)), and red bars show the results of the evaluation using GBCA-enhanced scans (pre- and post-contrast sequences: pre-contrast T1-weighted, T2-weighted, FLAIR, DWI/ADC, SWI (if present) + post-contrast T1-weighted). Comparison of agreements between GBCA-free and GBCA-enhanced predictions was significant for tumor grade (red star, *p* = 0.008), while it was insignificant for molecular marker (*p* > 0.05). Note: The interpretation of agreement values was as follows: 0.01–0.20, slight; 0.21–0.40, fair; 0.41–0.60, moderate; 0.61–0.80, substantial; and 0.81–0.99, almost perfect. GBCA, gadolinium-based contrast agent; IDH, isocitrate dehydrogenase

Group inter-rater agreement for tumor grade prediction was higher using GBCA-enhanced scans (0.68 (95%-CI: 0.58–0.78)) than using GBCA-free scans (0.56 (95%-CI: 0.46–0.66,) *p* = 0.008). Outcomes for molecular status prediction were substantial for both GBCA-free (0.75 (95%-CI: 0.69–0.80)) and GBCA-enhanced scans (0.77 (95%-CI: 0.71–0.82), *p* = 0.51); see Fig. 4.

Pairwise inter-rater agreements for tumor grade prediction were moderate or better for both GBCA-free (≥ 0.52 (95%-CI 0.38–0.65)) and GBCA-enhanced (≥ 0.59 (95%-CI 0.50–0.73)) scans. The results increased to substantial or almost perfect levels (≥ 0.74) after applying PABAK analysis, accounting for dataset imbalance. Outcomes for the molecular status prediction were substantial for both GBCA-free (≥ 0.73 (95%-CI 0.65–0.80)) and GBCA-enhanced (≥ 0.74 (95%-CI 0.66–0.81)) scans. Comparison analysis between GBCA-free and GBCA-enhanced agreements showed no significant differences (all *p* > 0.05) except for agreements in grade prediction for raters 1&2 (GBCA-free/GBCA-enhanced 0.53 (95%-CI: 0.40–0.66)/0.74 (95%-CI: 0.63–0.86), *p* = 0.003) and raters 1&3 (GBCA-free/GBCA-enhanced 0.52 (95%-CI: 0.38–0.65)/0.69 (95%-CI: 0.57–0.81), *p* = 0.02); see Table S6 and Fig. S9.

**Fig. 5:**
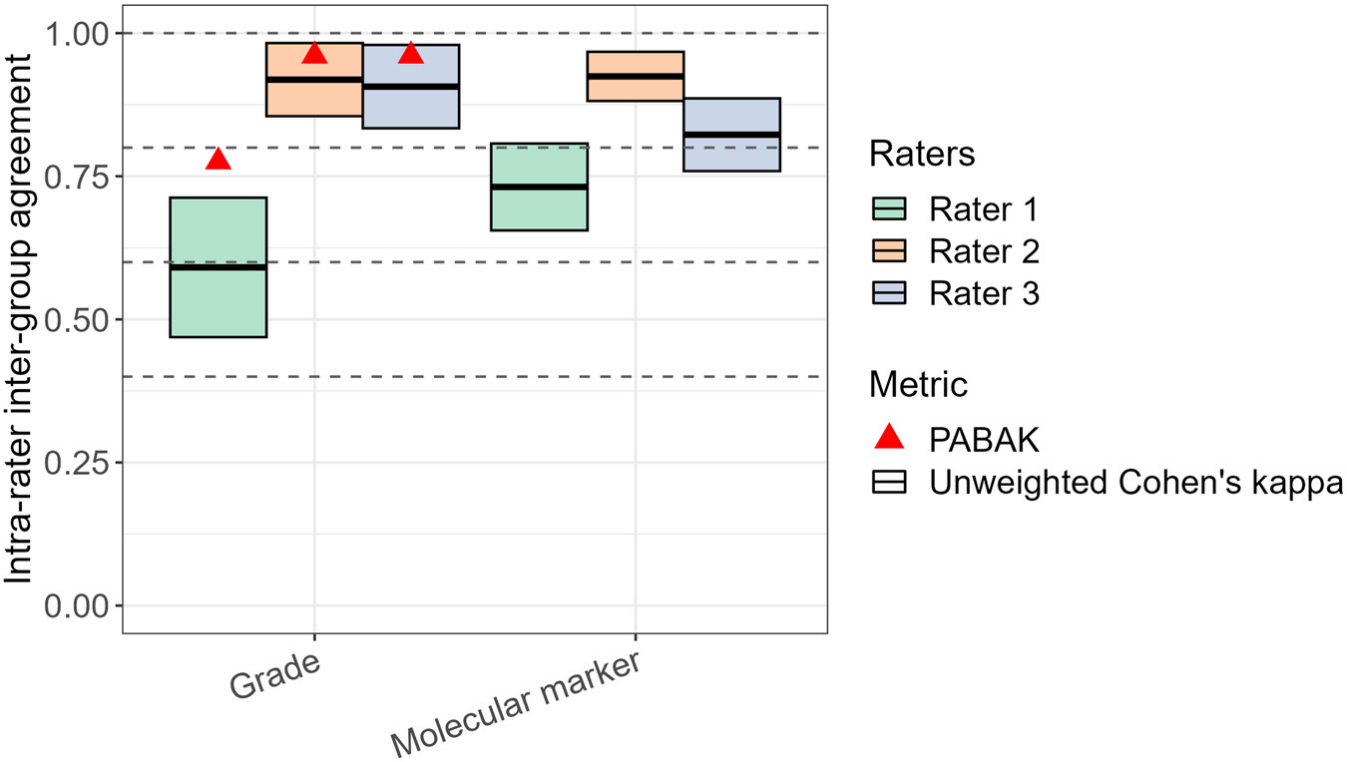
Intra-rater inter-group agreement in histomolecular diagnosis prediction of adult-type diffuse gliomas. The color box plot describes intra-rater inter-group agreement comparing GBCA-free and GBCA-enhanced histomolecular diagnosis predictions of each rater (rater 1 = green bar, rater 2 = orange bar, rater 3 = blue bar). Diagnosis predictions include tumor grade (low-grade: grade 2 vs. high-grade: grade 3/4) and molecular status (astrocytoma, IDH-mutant vs. oligodendroglioma, IDH-mutant and 1p/19q-codeleted vs. glioblastoma, IDH-wildtype) evaluations. Red triangles indicate prevalence-adjusted and bias-adjusted kappa (PABAK) values that compensate for the possible influence of dataset diagnosis imbalances. Comparison of agreements among all raters revealed significant differences for both tumor grade and molecular marker (*p* < 0.001). Note: The interpretation of agreement values was as follows: 0.01–0.20, slight; 0.21–0.40, fair; 0.41–0.60, moderate; 0.61–0.80, substantial; and 0.81–0.99, almost perfect. GBCA, gadolinium-based contrast agent; IDH, isocitrate dehydrogenase

Intra-rater inter-group agreements (Fig. 5) for grade prediction were moderate 0.59 (95%-CI: 0.47–0.71) for rater 1 and almost perfect for rater 2 (0.92 (95%-CI: 0.85–0.98)) and rater 3 (0.91 (95%-CI: 0.83–0.98)). Applying PABAK to account for diagnosis incidence imbalance further improved the results (raters 1/2/3: 0.78/0.96/0.96). The corresponding results for molecular status prediction were substantial for rater 1 (0.73 (95%-CI: 0.66–0.81)) and almost perfect for rater 2 (0.92 (95%-CI: 0.88–0.97)) and rater 3 (0.82 (95%-CI: 0.76–0.89)). The comparison of intra-rater agreements among all raters showed significant differences for both tumor grade (*p* < 0.001) and molecular status prediction (*p* < 0.001).

#### Agreement analysis for DPDT imaging features

**Fig. 6:**
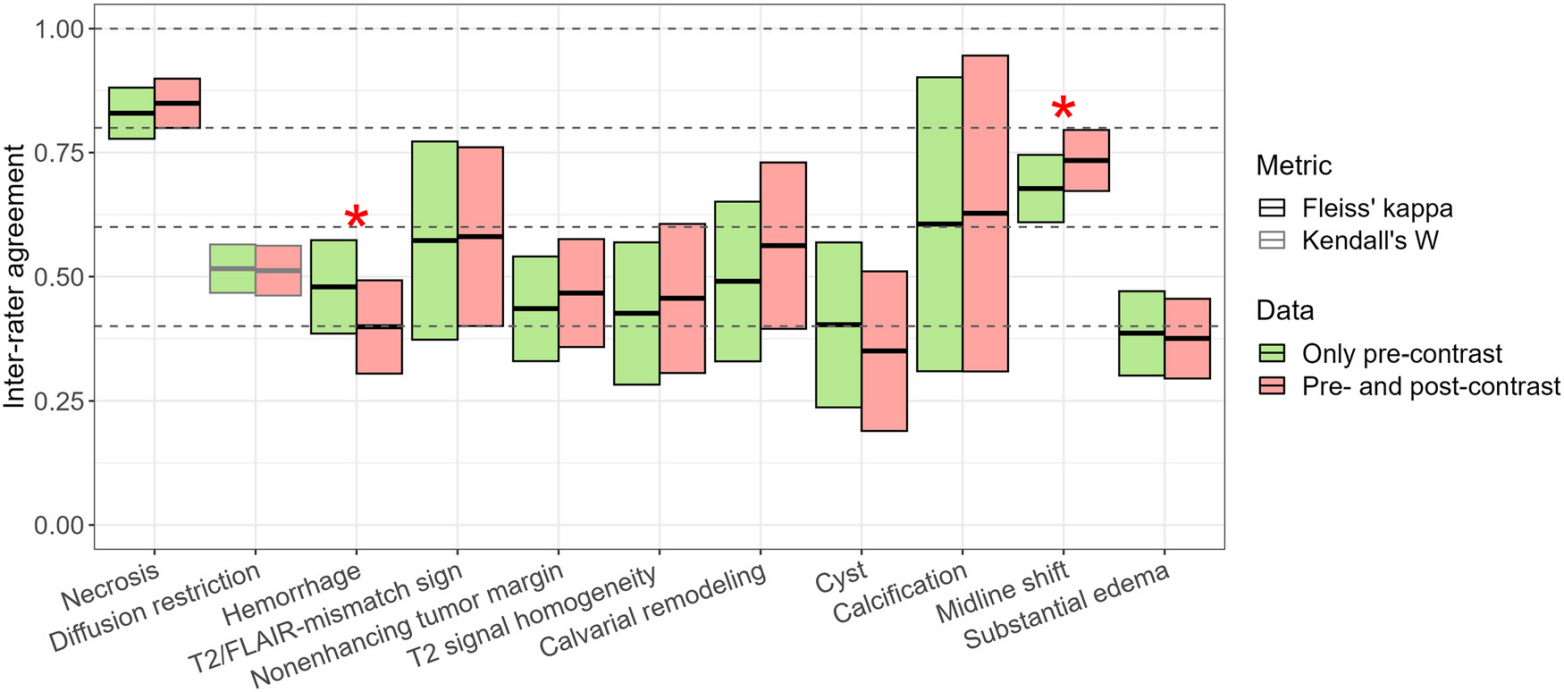
Group inter-rater agreement in evaluating imaging features included in the Diagnosis Prediction Decision tree (DPDT) for adult-type diffuse gliomas. The color box plot shows inter-rater agreement in evaluating single DPDT imaging features using GBCA-free or GBCA-enhanced scans among all raters. Green bars depict the results based on the assessment of GBCA-free scans (only pre-contrast sequences: pre-contrast T1-weighted, T2-weighted, FLAIR, DWI/ADC, and SWI (if present)), and red bars show the results of the evaluation using GBCA-enhanced scans (pre- and post-contrast sequences: pre-contrast T1-weighted, T2-weighted, FLAIR, DWI/ADC, SWI (if present) + post-contrast T1-weighted). Comparison of agreements between GBCA-free and GBCA-enhanced assessments was insignificant (*p* > 0.05) except for hemorrhage and midline shift (red stars, *p* = 0.02 and *p* = 0.04, respectively). Note: The interpretation of agreement values was as follows: 0.01–0.20, slight; 0.21–0.40, fair; 0.41–0.60, moderate; 0.61–0.80, substantial; and 0.81–0.99, almost perfect. GBCA, gadolinium-based contrast agent

Group inter-rater agreements were consistent (all *p* > 0.05) between the evaluation of GBCA-free and GBCA-enhanced scans except for hemorrhage and midline shift, showing significant differences (*p* = 0.02 and *p* = 0.04, respectively). The robust feature with almost perfect agreement was necrosis (≥ 0.83 (95%-CI: 0.78–0.88)). Calcification and midline shift showed substantial agreements (≥ 0.61 (95%-CI: 0.31–0.90)) while other features reached fair to moderate levels (0.35 (95%-CI: 0.19–0.51)–0.58 (95%-CI: 0.40–0.76)); see Table 4 and Fig. 6.

**Table 4:** Group inter-rater agreement in the evaluation of imaging features included in DPDT

Imaging features	GBCA-free scans	GBCA-enhanced scans
Necrosis^a^	0.83 (95%-CI: 0.78–0.88)	0.85 (95%-CI: 0.80–0.90)
Diffusion restriction^b^	0.52 (95%-CI: 0.47–0.56)	0.51 (95%-CI: 0.46–0.56)
Hemorrhage^a,c^	0.48 (95%-CI: 0.39–0.57)	0.40 (95%-CI: 0.31–0.49)
T2-FLAIR mismatch sign^a^	0.57 (95%-CI: 0.37–0.77)	0.58 (95%-CI: 0.40–0.76)
Non-enhancing tumor margin^a^	0.44 (95%-CI: 0.33–0.54)	0.47 (95%-CI: 0.36–0.58)
T2 homogeneity^a^	0.43 (95%-CI: 0.28–0.57)	0.46 (95%-CI: 0.31–0.61)
Calvarial remodeling^a^	0.49 (95%-CI: 0.33–0.65)	0.56 (95%-CI: 0.39–0.73)
Cyst^a^	0.40 (95%-CI: 0.24–0.57)	0.35 (95%-CI: 0.19–0.51)
Calcification^a^	0.61 (95%-CI: 0.31–0.90)	0.63 (95%-CI: 0.31–0.95)
Midline shift^a,c^	0.68 (95%-CI: 0.61–0.75)	0.73 (95%-CI: 0.67–0.80)
Substantial edema^a^	0.39 (95%-CI: 0.30–0.47)	0.38 (95%-CI: 0.30–0.46)

Table 4 shows the group inter-rater agreement results for imaging features involved in DPDT using both GBCA-free and GBCA-enhanced scans

Note: Interpretation of agreement values was as follows: 0.01–0.20, slight; 0.21–0.40, fair; 0.41–0.60, moderate; 0.61–0.80, substantial; and 0.81–0.99, almost perfect

*DPDT* diagnosis prediction decision tree, *GBCA* gadolinium-based contrast agent, *GBCA*-*free scans* pre-contrast T1-weighted, T2-weighted, FLAIR, DWI/ADC, SWI (if present) sequences, *GBCA-enhanced scans* pre-contrast T1-weighted, T2-weighted, FLAIR, DWI/ADC, SWI (if present) + post-contrast T1-weighted sequences

^a^ Fleiss’ kappa

^b^ Kendall’s *W*

^c^ Features with significant difference (*p* < 0.05) between GBCA-free and GBCA-enhanced agreement values

Pairwise inter-rater agreements were at least substantial for necrosis (≥ 0.80 (95%-CI: 0.73–0.87)) with further improvement after applying PABAK analysis (≥ 0.82). The outcomes for other features varied between 0.13 (95%-CI: −0.04–0.31) and 0.80 (95%-CI: 0.52–1.07). However, the results increased with PABAK analysis (range: 0.50–94), showing the impact of dataset imbalance. There were no significant differences (all *p* > 0.05) between GBCA-free and GBCA-enhanced agreements with a few exceptions (rater 1&3: cyst *p* = 0.04, midline shift *p* = 0.03, rater 1&2: midline shift *p* = 0.02); see Table S7 and Fig. S10.

Intra-rater agreements were almost perfect for necrosis (≥ 0.82 (95%-CI: 0.75–0.89)) and midline shift (≥ 0.81 (95%-CI 0.73–0.89)), substantial or better for hemorrhage, T2-FLAIR mismatch sign, calvarial remodeling, calcification and substantial edema (≥ 0.64 (95%-CI: 0.44–0.84)), fair or better for other features (≥ 0.35 (95%-CI: 0.18–0.52)). The comparison of intra-rater agreements among all raters showed significant differences (all *p* < 0.05) except for the T2-FLAIR mismatch sign (*p* = 0.93) and calcification (*p* = 0.96); see Table 5 and Fig. S11.

**Table 5:** Intra-rater inter-group agreement in the evaluation of imaging features included in DPDT

Imaging features	Rater 1	Rater 2	Rater 3
Necrosis^a^ *PABAK*	0.82 (95%-CI: 0.75–0.89) *0.84*	0.91 (95%-CI: 0.87–0.96) *0.92*	0.83 (95%-CI: 0.77–0.90) *0.85*
Diffusion restriction^b^	0.56 (95%-CI: 0.49–0.64)	0.75 (95%-CI: 0.67–0.82)	0.76 (95%-CI: 0.70–0.82)
Hemorrhage^a^ *PABAK*	0.69 (95%-CI: 0.59–0.80) *0.80*	0.87 (95%-CI: 0.81–0.93) *0.89*	0.87 (95%-CI: 0.77–0.97) *0.96*
T2-FLAIR mismatch sign^a^ *PABAK*	0.76 (95%-CI: 0.52–1.00) *0.97*	1.00 (95%-CI: 1.00–1.00) *1.00*	0.68 (95%-CI: 0.45–0.91) *0.95*
Non-enhancing tumor margin^a^ *PABAK*	0.35 (95%-CI: 0.18–0.52) *0.78*	0.85 (95%-CI: 0.77–0.94) *0.93*	0.66 (95%-CI: 0.54–0.77) *0.80*
T2 homogeneity^a^ *PABAK*	0.45 (95%-CI: 0.30–0.60) *0.78*	0.90 (95%-CI: 0.79–1.00) *0.97*	0.66 (95%-CI: 0.50–0.83) *0.91*
Calvarial remodeling^a^ *PABAK*	0.64 (95%-CI: 0.44–0.84) *0.92*	0.95 (95%-CI: 0.88–1.00) *0.99*	0.72 (95%-CI: 0.51–0.93) *0.95*
Cyst^a^ *PABAK*	0.40 (95%-CI: 0.21–0.59) *0.81*	0.74 (95%-CI: 0.55–0.93) *0.95*	0.55 (95%-CI: 0.28–0.82) *0.94*
Calcification^a^ *PABAK*	0.75 (95%-CI: 0.48–01.00) *0.90*	1.00 (95%-CI: 1.00–1.00) *1.00*	0.87 (95%-CI: 0.61–1.00) *0.97*
Midline shift^a^ *PABAK*	0.81 (95%-CI: 0.73–0.89) *0.84*	0.91 (95%-CI: 0.86–0.96) *0.91*	0.83 (95%-CI: 0.77–0.89) *0.83*
Substantial edema^a^ *PABAK*	0.65 (95%-CI: 0.55–0.74) *0.70*	0.78 (95%-CI: 0.68–0.89) *0.89*	0.84 (95%-CI: 0.77–0.90) *0.83*

Table 5 shows the intra-rater inter-group agreement results for imaging features involved in DPDT, comparing the evaluation of GBCA-free scans with GBCA-enhanced scans for each rater

The interpretation of agreement kappa values was as follows: 0.01–0.20, slight; 0.21–0.40, fair; 0.41–0.60, moderate; 0.61–0.80, substantial; and 0.81–0.99, almost perfect. Italicized PABAK stands for prevalence-adjusted bias-adjusted kappa adjusting the kappa for imbalances caused by differences in the prevalence and bias. The agreement level interpretation ranges are like for standard kappa.

*DPDT* diagnosis prediction decision tree, *GBCA* gadolinium-based contrast agent, *GBCA-free scans* pre-contrast T1-weighted, T2-weighted, FLAIR, DWI/ADC, SWI (if present) sequences, *GBCA-enhanced scans* pre-contrast T1-weighted, T2-weighted, FLAIR, DWI/ADC, SWI (if present) + post-contrast T1-weighted sequences

^a^ Unweighted Cohen’s kappa

^b^ Weighted Cohen’s kappa

## Discussion

In this study, we developed a DPDT, incorporating eleven imaging features from conventional GBCA-free MRI for adult-type diffuse gliomas. DPDT, assessed by three raters with variable levels of experience, demonstrated a high predictive performance for the classification of both tumor grade (accuracy ≥ 0.85 (95%-CI: 0.80–0.88)) and molecular status (overall accuracy ≥ 0.75 (95%-CI: 0.70–0.80). Adding GBCA-enhanced images to the evaluation showed comparable results (accuracy ≥ 0.87 (95%-CI: 0.82–0.90) and overall accuracy ≥ 0.77 (95%-CI: 0.71–0.81), respectively). Comparison of GBCA-free and GBCA-enhanced outcomes (accuracy, sensitivity, and specificity) revealed insignificant differences except for rater 1’s sensitivity in grade prediction (GBCA-free/GBCA-enhanced = 0.90/0.95, *p* = 0.006).

Our study suggests that the proposed DPDT using GBCA-free MRI could be as reliable as standard GBCA-enhanced MRI in preoperative diagnostic glioma assessment. Previous studies often evaluated the diagnostic efficacy of conventional MRI, including GBCA-enhanced images, making it challenging to determine the complementary role of GBCA. For instance, Du et al [31] and Setyawan et al [20] proposed preoperative glioma grading (AUC: 0.93 and 1.00, respectively) and IDH genotyping (AUC: 0.86 and 0.93, respectively) models incorporating enhancement features alongside other GBCA-free imaging features such as hemorrhage or cysts. Although a recent study [32] proposed an MRI scoring system utilizing GBCA-free features, it specifically assessed non-enhancing gliomas. However, focusing exclusively on GBCA-free imaging features without excluding enhancing gliomas could help to better comprehend the additional benefit of GBCA in decision-making. Our study addresses this gap by assessing a large glioma cohort through a head-to-head comparison of GBCA-free and GBCA-enhanced MRI, ultimately refuting the additive value of GBCA. The evaluation across different patient subgroups, such as age or tumor location, was similar for GBCA-free and GBCA-enhanced evaluations, stressing the limited added value of GBCA-enhanced images. The subgroup analysis revealed that DPDT performs better in older age groups, likely due to the high prevalence of glioblastoma in this demographic, which often exhibits typical imaging features such as necrosis, facilitating identification. The performance was less accurate for tumors in the thalamus, possibly because thalamic lesions display more distinct imaging features than hemispheric tumors. Importantly, inter-rater agreement regarding histomolecular diagnosis was not improved by GBCA use, highlighting the potential value of GBCA-free DPDT in real-world clinical settings, while the IDH prediction accuracy of only 77%, even with GBCA indicates limitations for radiology in general.

Our DPDT algorithm comprises eleven imaging features, each correlated with the respective histomolecular glioma diagnosis in previous studies [18–25]. Among these, necrosis, a glioblastoma biomarker in DPDT, was the robust feature, demonstrating at least substantial inter-/intra-rater agreement (≥ 0.80 (95%-CI: 0.73–0.87)). A recent study [33] investigating the reliability of imaging-based necrosis found a strong agreement between this and pathological necrosis (0.77 (95%-CI: 0.64–0.90)). That study observed a significant correlation between imaging-based necrosis and tumor grade as well as IDH status (*p* < 0.001), alongside substantial inter-rater agreement (0.67 (95%-CI: 0.49–0.85)), comparable to our study. However, their necrosis assessment relied on GBCA-enhanced MRI, similar to most previous studies [18–20]. Conversely, our evaluation of necrosis utilized both GBCA-free and GBCA-enhanced MRI, revealing almost perfect intra-rater agreements (≥ 0.82 (95%-CI: 0.75–0.89)), thus underscoring the efficacy of GBCA-free MRI in this context. Among other DPDT features, varying levels of inter- and intra-rater agreement (range: 0.13 (95%-CI: – 0.04 to 0.31)–1.00 (95%-CI: 1.00–1.00)) were observed. T2-FLAIR mismatch sign (≥ 0.51 (95%-CI: 0.23–0.80)) and calcification (≥ 0.49 (95%-CI: 0.08–0.89)) emerged as the most consistent imaging features associated with astrocytoma and oligodendroglioma, respectively, in DPDT. Group inter-rater agreements for hemorrhage and midline shift differed significantly between GBCA-free and GBCA-enhanced evaluations. The discrepancy in hemorrhage assessment may stem from inconsistent SWI availability and varying degrees of pre-contrast T1 hyperintensity, rather than from the availability of GBCA-enhanced images. Similarly, for the midline shift, the disagreement could result from the prevalence of cases with a minimal shift around the 5 mm threshold, leading to measurement variations between evaluations and raters. Utilizing the decision tree enables a systematic approach to imaging assessment, potentially improving diagnostic thoroughness, as less experienced raters demonstrated comparable or better performance to experienced radiologists. Beyond assessment by radiologists, the present study may deliver insights for researchers focusing on AI-based algorithms, as DPDT provides imaging characteristics relevant to algorithm decision-making.

This study has several limitations demanding future research. The study focuses on a specific scenario, and a relatively small sample size rated by only two observers was used to develop  the DPDT. Besides the scientific outcomes, the study’s clinical value is potentially limited to situations where tissue diagnosis is not feasible, e.g., due to poor clinical condition, tumor location or when GBCA administration is contraindicated or not desired. Moreover, the omission of clinical factors such as age and the lack of a longitudinal evaluation in DPDT will have impacted diagnostic predictions. Additionally, despite IDH-wildtype diffuse gliomas being classified as HGG in this study regardless of histological grade, recent studies suggest that histologically grade 2 IDH-wildtype diffuse gliomas with isolated telomerase reverse transcriptase (TERT) promoter mutation may exhibit a more favorable outcome than glioblastoma, IDH-wildtype [34]. Further work is needed to incorporate clinical factors and other imaging biomarkers, including temporal imaging evaluation, and extend DPDT to other brain tumor differentials, such as metastasis or lymphoma, and non-tumor lesions, such as demyelination or infection. Another significant limitation is the exclusion of perfusion data due to the inconsistent availability of ASL data for comparison with the standard method of dynamic susceptibility contrast (DSC)-MRI. Provided a head-to-head comparison can be guaranteed, perfusion should be examined in a future DPDT study. Moreover, an extended consecutive study should incorporate time-tracking of the DPDT usage to evaluate its clinical utility compared to standard evaluation.

In conclusion, the proposed decision tree enables non-invasive preoperative diagnosis of adult-type diffuse gliomas using only GBCA-free MRI, regardless of the rater’s experience level. Future research should develop a generalized decision tree with diverse brain mass lesions and advanced imaging techniques and test it with additional raters and new data.

## Supplementary information

ELECTRONIC SUPPLEMENTARY MATERIAL

## Abbreviations

DPDT: Diagnosis prediction decision tree
GBCA: Gadolinium-based contrast agent
IDH: Isocitrate dehydrogenase
VASARI: Visually AcceSAble Rembrandt Images
