# Supplementary material for: Preoperative prediction of diffuse glioma type and grade in adults: a gadolinium-free MRI-based decision tree
Source: Eur Radiol. 2024 Oct 19;35(3):1242–54. doi: 10.1007/s00330-024-11140-5 (PMC11836213; doi:10.1007/s00330-024-11140-5)
Supplement: Supplementary file 1 — ELECTRONIC SUPPLEMENTARY MATERIAL [file 330_2024_11140_MOESM1_ESM.pdf]

Preoperative Prediction of Diffuse Glioma Type and Grade in Adults: a Gadolinium-Free MRI-Based Decision Tree

ELECTRONIC SUPPLEMENTARY MATERIAL

Eur Radiol (2024) Azizova A, Prysiazhniuk Y, Wamelink IJHG, et al.

Table of Contents

Rationale for the selection of DPDT imaging features **2**
Explanation of the Diagnosis Prediction Decision Tree (DPDT) **4**
Guide material for the raters **5**
Supplementary Tables **8**
Table S1. MRI scan parameters 8
Table S2. Imaging features involved in the Diagnosis Prediction Decision Tree 10
Table S3. Prediction performance of the raters in the development dataset 11
Table S4. Prediction performance of the raters in the optimization dataset 13
Table S5. Failed GBCA-free and GBCE-enhanced diagnosis predictions across various tumor locations 15
Table S6. Pairwise inter-rater agreement in the prediction of the histomolecular diagnosis 16
Table S7. Pairwise inter-rater agreement in the evaluation of imaging features included in DPDT 17
Supplementary Figures and Figure Captions **19**
Figure S1. Case examples for the evaluation of necrosis and diffusion. 19
Figure S2. Case examples for the evaluation of hemorrhage. 20
Figure S3. Case examples for the evaluation of T2-FLAIR mismatch sign, non-enhancing tumor margin, and T2 signal homogeneity. 21
Figure S4. Case examples for the evaluation of cyst and calcification. 22
Figure S5. Case examples for the evaluation of calvarial remodeling, midline shift, and substantial edema. 23
Figure S6. Per-rater confusion matrices for predicting histomolecular diagnosis of adult-type diffuse gliomas using GBCA-free and GBCA-enhanced scans in the development dataset. 24
Figure S7. Per-rater confusion matrices for predicting histomolecular diagnosis of adult-type diffuse gliomas using GBCA-free and GBCA-enhanced scans in the optimization dataset. 25
Figure S8. Per-rater confusion matrices for predicting histomolecular diagnosis of adult-type diffuse gliomas using GBCA-free and GBCA-enhanced scans in the test dataset. 26
Figure S9. Pairwise inter-rater agreement in histomolecular diagnosis prediction of adult-type diffuse gliomas using GBCA-free vs. GBCA-enhanced scans. 27
Figure S10. Pairwise inter-rater agreement in the evaluation of imaging features included in the Diagnosis Prediction Decision tree (DPDT) for adult-type diffuse gliomas using GBCA-free vs. GBCA-enhanced scans. 28
Figure S11. Intra-rater inter-group agreement in the evaluation of imaging features included in the Diagnosis Prediction Decision tree (DPDT) for adult-type diffuse gliomas. 29

Rationale for the selection of DPDT imaging features

The selection of specific imaging features for DPDT was primarily guided by the clinical experience of the raters, who also referenced established imaging biomarkers [1-8] commonly used in clinical practice.

Glioblastoma, IDH-wildtype:

Necrosis and hemorrhage are well-recognized imaging biomarkers of glioblastoma, IDH-wildtype. However, their diffusion properties can still identify tumors without these characteristic features. Glioblastomas, particularly those imaged before necrosis occurs, often show diffusion restriction. As a result, these three features were considered independent imaging biomarkers of glioblastoma, IDH-wildtype, in DPDT.

Astrocytoma, IDH-mutant:

Astrocytomas exhibit a range of imaging features. T2-FLAIR mismatch sign is considered the most reliable biomarker, with studies showing up to 100% specificity. Tumors displaying this sign typically have a homogeneous T2 signal and well-defined tumor margins. However, not all astrocytomas with homogeneous T2 signal or well-defined margins exhibit the T2-FLAIR mismatch sign. Thus, these three features were treated as independent biomarkers for astrocytomas. Although astrocytomas with these features are often low-grade, the presence of a midline shift or substantial edema may suggest an aggressive, high-grade tumor. High-grade astrocytomas may also display heterogeneous T2 signal and ill-defined margins. In such cases, after excluding the imaging features of oligodendrogliomas, as discussed below, a diagnosis of high-grade astrocytoma should be considered.

Oligodendroglioma, IDH-mutant, and 1p/19q-codeleted:

Oligodendrogliomas typically have a better prognosis but present with a heterogeneous appearance on MRI. Tumors with T2 heterogeneity or ill-defined borders should be evaluated for the possibility of 1p/19q codeletion. Supporting imaging features such as calvarial remodeling, cyst, or calcification are well-documented in the literature and commonly used in clinical practice. Thus, these three imaging features were considered as a oligodendroglioma biomarkers in tumors with T2 heterogeneity and ill-defined borders in DPDT. While determining the grade of oligodendrogliomas can be challenging, the presence of a midline shift or substantial edema may suggest a higher grade.

1. Lasocki A, Buckland ME, Drummond KJ et al (2022) Conventional MRI features can predict the molecular subtype of adult grade 2-3 intracranial diffuse gliomas. Neuroradiology 64:2295–2305
2. Nam YK, Park JE, Park SY et al (2021) Reproducible imaging-based prediction of molecular subtype and risk stratification of gliomas across different experience levels using a structured reporting system. Eur Radiol 31:7374–7385
3. Setyawan NH, Choridah L, Nugroho HA et al (2024) Beyond invasive biopsies: using VASARI MRI features to predict grade and molecular parameters in gliomas. Cancer Imaging 24:3
4. Çelik S, Öven BB, Demir MK et al (2021) Magnetic resonance imaging criteria for prediction of isocitrate dehydrogenase (IDH) mutation status in patients with grade II-III astrocytoma and oligodendroglioma. Clin Neurol Neurosurg 207:106745
5. Corell A, Ferreyra Vega S, Hoefling N et al (2020) The clinical significance of the T2-FLAIR mismatch sign in grade II and III gliomas: a population-based study. BMC Cancer 20:450
6. Han Z, Chen Q, Zhang L et al (2022) Radiogenomic association between the T2-FLAIR mismatch sign and IDH mutation status in adult patients with lower-grade gliomas: an updated systematic review and meta-analysis. Eur Radiol 32:5339–5352
7. Smits M (2016) Imaging of oligodendroglioma. Br J Radiol 89:20150857
8. Johnson DR, Diehn FE, Giannini C et al (2017) Genetically Defined Oligodendroglioma Is Characterized by Indistinct Tumor Borders at MRI. AJNR Am J Neuroradiol 38:678–684

Explanation of the Diagnosis Prediction Decision Tree (DPDT)

DPDT incorporates seven VASARI (necrosis, diffusion restriction, hemorrhage, non-enhancing tumor margins, calvarial remodeling, cysts, and proportion of edema) and four non-VASARI imaging features (T2-FLAIR mismatch sign, T2 signal homogeneity, calcification, and midline shift). Initially, necrosis, diffusion restriction (visually assessed on both b-1000 and ADC maps), or hemorrhage serve as independent imaging biomarkers for glioblastoma, IDH-wildtype. In cases where these biomarkers are absent, the subsequent evaluation focuses on the T2/FLAIR mismatch sign, well-defined non-enhancing tumor margin, or T2 signal homogeneity. The presence of any of these features independently marks astrocytoma, IDH-mutant, while the absence prompts assessment of calvarial remodeling, cysts, or calcification. The presence of these latter features suggests oligodendroglioma, IDH-mutant and 1p/19q-codeleted, whereas their absence indicates high-grade astrocytoma, IDH-mutant. The midline shift or substantial edema is the chosen imaging feature for determining the histological grade of astrocytomas or oligodendrogliomas, as these features correlate with high-grade gliomas. Refer to Figure 1 for an illustration of DPDT.

Guide material for the raters

Definition of single imaging features used in Diagnosis Prediction Decision Tree (DPDT)

- **Necrosis, modified VASARI feature 7* (yes, no); see Figure S1 (a-c)**
  - area with irregular and/or thick margins and the following internal characteristics: T1 hypointensity, T2 hyperintensity, and high ADC values like fluid
  - should not be cysts, clusters of microcysts, or enlarged perivascular space
- **Diffusion restriction, VASARI feature 17* (yes: restricted or no: dubious/facilitated); see Figure S1 (d-i)**
  - restricted- characterized by high signal intensity on TRACE/DWI and notably low ADC values compared to the normal brain parenchyma
  - dubious/facilitated- characterized by high signal intensity on TRACE/DWI with corresponding ADC values similar to the normal brain parenchyma (dubious) or high/low signal intensity on TRACE/DWI with ADC values notably higher than the typical brain parenchyma (facilitated)
  - the areas featuring reduced ADC signal intensity due to necrotic or hemorrhagic tumor components should be disregarded
- **Hemorrhage, VASARI feature 16* (yes, no); see Figure S2**
  - yes- (a) the presence of drop-out or blooming on post-processed SWI images exhibiting identical signal intensity to internal cerebral veins/superior sagittal sinus on filtered phase images or (b) any intrinsic hyperintense foci in proximity to or within the necrotic region of the tumor on pre-contrast T1-weighted images should also be recognized as hemorrhage
  - no- (a) the absence of drop-out or blooming on post-processed SWI images or the presence of drop-out or blooming with a signal intensity opposite to internal cerebral veins/superior sagittal sinus, indicative of calcification on filtered phase images, or (b) when observations are unclear on pre-contrast T1-weighted images or may potentially signify mineral presence rather than hemorrhage
- **T2-Flair mismatch sign (yes, no); see Figure S3 (a,b)**
  - yes- the presence of complete/near complete homogeneous hyperintense signal on T2-weighted images with relatively homogeneous or heterogeneous

hypointense signal in most of these regions (> 90%) on T2-FLAIR, except for a hyperintense complete or incomplete peripheral rim
    - no- the presence of heterogeneous signal intensity on T2-weighted images and/or the lack of suppressed T2 signal on FLAIR, including the absence of a hyperintense peripheral rim

- **Non-enhancing tumor margin, VASARI feature 13* (well-defined, ill-defined); see Figure S3 (c,d)**
    - well-defined- tumor margins should be deemed well-defined when they can be easily followed consistently across nearly the entire tumor, encompassing more than 90% of its volume.
    - ill-defined- indistinct or blurred margins, or margins that align with white matter tracts and are challenging to distinguish from surrounding edema across most of the tumor volume.
- **T2 signal homogeneity (yes/homogeneous, no/heterogeneous); see Figure S3 (c,d)**
    - homogeneous- almost the same signal intensity throughout the tumor except for the lesion rim, vessels (dark dots or lines), cysts, perivascular spaces, and probably infiltrated but normal-appearing cortex compared to the other tumor parts.
    - heterogeneous- mainly different signal intensity, including hypointense, isointense, and/or hyperintense signal compared to normal brain cortex, throughout the tumor
- **Cyst, VASARI feature 8* (yes, no); see Figure S4 (a-c)**
    - well-defined, rounded, often eccentric regions of high T2-weighted signal and low T1-weighted signal essentially resembling the signal intensity of cerebrospinal fluid, with very thin, uniform, smooth, non-enhancing or regularly enhancing walls, possibly with thin, regular, internal septations
- **Calcification (yes, no); see Figure S4 (d-h)**
    - yes- the presence of drop-out or blooming on post-processed SWI images with a signal intensity opposite to that of internal cerebral veins/superior sagittal sinus on filtered phase images. When present, the calcified choroid plexus or pineal gland serves as an internal reference for calcification; a similar signal intensity to these structures suggests calcification rather than hemorrhage.

  - no- the absence of drop-out or blooming on post-processed SWI images or the presence of drop-out or blooming with the same signal intensity as internal cerebral veins/superior sagittal sinus, indicative of hemorrhage on filtered phase images.
- **Calvarial remodeling, VASARI feature 25* (yes, no); see Figure S5 (a,b)**
  - yes- the presence of the evident erosion or remodeling of the inner table of the skull, possibly indicative of the gradual growth of the tumor.
  - no – the lack of observable bone erosion or remodeling near the tumor or the tumor is far from the calvarial bones.
- **Midline shift (yes, no); see Figure S5 (c)**
  - yes- the presence of more than 5 mm shift of the midline structures to the left or right according to the line drawn coplanar with falx connecting the anterior and posterior superior sagittal sinus attachments
  - no- the absence of a midline shift or the presence of a midline shift measuring 5 mm or less, based on the line drawn coplanar with the falx connecting the anterior and posterior superior sagittal sinus attachments.
- **Substantial edema, modified VASARI feature 14 (yes, no); see Figure S5 (d)**
  - yes- significant edema is defined as equal to or exceeding half of the tumor volume (≥ 50%). The signal intensity of edema surrounding the tumor should surpass that of the non-enhancing tumor and be lower than that of cerebrospinal fluid. Pseudopods are indicative of edema.
  - no- the absence of edema surrounding the tumor or the presence of edema constituting < 50% of the tumor volume.

* *VASARI features according to VASARI Research Project*

https://wiki.cancerimagingarchive.net/display/Public/VASARI+Research+Project.

Supplementary Tables

Table S1. MRI scan parameters

Sequence parameters	Scanners						
	GE 1.5T Signa HDxt	***GE 3T DISCOVERY MR750***	***Philips 1.5T Achieva***	***Philips 3T Ingenuity***	***Siemens 1.5T Avanto***	***Siemens 3T MAGNETOM Vida***	***Toshiba 3T Titan3T***
2D T1-weighted							
plane	axial	axial	-	axial	axial	-	axial
TE; ms, min-max	9-12	7.9-9.7	-	10	7.8-17	-	8
TR; ms, min-max	520-600	600-731	-	599	500-718	-	550
FA; min-max	90	90-125	-	70	90	-	80
3D T1-weighted							
plane	-	axial	axial	axial	axial	sagittal	-
TE; ms, min-max	-	2	3.3-4.6	3	4.5-11	2.3	-
TR; ms, min-max	-	4.7	6.5-8.7	7	700-2,700	2,300	-
TI; ms, min-max	-	650	-	-	0-950	900	-
FA; min-max	-	15	8-10	12	8-120	8	-
T2-weighted							
plane	axial	axial	axial	axial	axial	axial	axial
TE; ms, min-max	98-104	82-88	100-110	85	93-104	74	90
TR; ms, min-max	4,376-4,840	4,889-6,872	3,404-5,251	2,767-3,182	2,830-5,562	4,100-6,280	5,500-5,526
FA; min-max	90	90-111	90	90	150-180	150	90
2D FLAIR							
plane	axial	-	axial	-	axial	-	-
TE; ms, min-max	118-120	-	140	-	88-109	-	-
TR; ms, min-max	9,002-9,502	-	9,000-11,000	-	8,870-9,000	-	-
TI; ms, min-max	2,250	-	2,800	-	2,500	-	-
FA; min-max	90	-	90	-	150	-	-
3D FLAIR							
plane	sagittal	sagittal	axial	sagittal	sagittal	sagittal	axial
TE; ms, min-max	96-122	126-135	286-306	279	334	388-430	451
TR; ms, min-max	6,000-6,500	8,000-8,002	4,800	4,800	6,500	5,000-7,700	5,600
TI; ms, min-max	1,925-1,987	2,331-2,347	1,660	1,650	2,200	1,650-2,400	1,900
FA; min-max	90	90	90	90	120	120	90
DWI							
plane	axial	axial	axial	axial	axial	axial	axial
TE; ms, min-max	81-105	62-87	72-119	74-97	90-122	68	82
TR; ms, min-max	8,000-8,500	4,000-7,200	2,674-6,448	3,496-6,354	3,400-10,500	3,200	7,500
FA; min-max	90	90	90	90	90	90	90
b value	1,000	1,000	1,000	1,000	1,000	1,000	1,000
SWI							
plane	-	axial	-	axial	axial	axial	-
TE; ms, min-max	-	25	-	25-30	40	25	-

TR; ms, min-max	-	31	-	18-21	49	31	-
FA; min-max	-	15	-	10	15	15	-
2D post-contrast T1-weighted							
plane	axial	axial	-	axial	axial	-	axial
TE; ms, min-max	9-12	7.9-8.4	-	10	8.7-17	-	8
TR; ms, min-max	520-600	600-650	-	599	550-718	-	550
FA; min-max	90	90-125	-	70	90	-	80
3D post-contrast T1-weighted							
plane	axial	axial	axial	axial	sagittal	axial	axial
TE; ms, min-max	3-5	2-3.2	3.3-4.6	3	2.9-4.5	2.3	2.4
TR; ms, min-max	8-13	4.6-8.3	6.7-8.7	7	1,900-2,700	2,300	5.7
TI; ms, min-max	0-450	450-650	-	-	950-1,100	900	900
FA; min-max	12	15	8-10	12	8	8	9

Caption: Table S1 describes the MRI acquisition parameters for all seven scanners.

Abbreviations: 2D/3D = 2/3-dimensional, DWI = diffusion-weighted imaging, FA = flip angle, FLAIR = fluid-attenuated inversion recovery, max = maximum value, min = minimum value, ms = millisecond, 1.5T/3T = 1.5 tesla/3 tesla, TE = time of echo, TI = time of inversion, TR = time of repetition, SWI = susceptibility-weighted imaging

Note: *The evaluation of MRI sequences while assigning ratings was done in the axial plane. When necessary, 3D sagittal scans were reformatted to the axial plane.*

All values are extracted from the DICOM headers.

Table S2. Imaging features involved in the Diagnosis Prediction Decision Tree		
**Seven VASARI features (Vf)**		**Four non-VASARI features**
Presence of necrosis	modified Vf 7	T2-FLAIR mismatch sign
Diffusion restriction	Vf 17	T2 signal homogeneity
Hemorrhage	Vf 16	Calcification
Non-enhancing tumor margin	Vf 13	Midline shift
Calvarial remodeling	Vf 25	
Cyst	Vf 8	
Substantial edema	modified Vf 14	

Caption: Table S2 describes seven VASARI and four non-VASARI imaging features involved in the Diagnosis Prediction Decision Tree (DPDT) for grade 2-4 adult-type diffuse gliomas.

Table S3. Prediction performance of the raters in the development dataset			
**Results**		**Rater 1**	**Rater 2**
**Histopathological grade with GBCA-free MRI**			
Accuracy		0.95	0.89
Sensitivity		0.97	0.91
Specificity		0.80	0.80
Positive predictive value		0.97	0.97
Negative predictive value		0.80	0.57
**Histopathological grade with GBCA-enhanced MRI**			
Accuracy		0.97	0.87
Sensitivity		1.00	0.88
Specificity		0.80	0.80
Positive predictive value		0.97	0.97
Negative predictive value		1.00	0.50
**Molecular diagnosis with GBCA-free MRI**			
Overall accuracy *(astrocytoma, IDH-mutant vs. oligodendroglioma, IDH-mutant and 1p/19q-codeleted vs. glioblastoma, IDH-wildtype)*		0.76	0.74
Accuracy	Astrocytoma, IDH-mutant	0.76	0.82
	Oligodendroglioma, IDH-mutant and 1p/19q-codeleted	0.95	0.89
	Glioblastoma, IDH-wildtype	0.82	0.76
Sensitivity	Astrocytoma, IDH-mutant	0.60	0.80
	Oligodendroglioma, IDH-mutant and 1p/19q-codeleted	1.00	1.00
	Glioblastoma, IDH-wildtype	0.78	0.72
Specificity	Astrocytoma, IDH-mutant	0.79	0.82
	Oligodendroglioma, IDH-mutant and 1p/19q-codeleted	0.95	0.89
	Glioblastoma, IDH-wildtype	1.00	1.00
Positive predictive value	Astrocytoma, IDH-mutant	0.30	0.40
	Oligodendroglioma, IDH-mutant and 1p/19q-codeleted	0.33	0.20
	Glioblastoma, IDH-wildtype	1.00	1.00
Negative predictive value	Astrocytoma, IDH-mutant	0.93	0.97
	Oligodendroglioma, IDH-mutant and 1p/19q-codeleted	1.00	1.00
	Glioblastoma, IDH-wildtype	0.46	0.40
**Molecular diagnosis with GBCA-enhanced MRI**			
Overall accuracy *(astrocytoma, IDH-mutant vs. oligodendroglioma, IDH-mutant and 1p/19q-codeleted vs. glioblastoma, IDH-wildtype)*		0.82	0.84
Accuracy	Astrocytoma, IDH-mutant	0.82	0.87
	Oligodendroglioma, IDH-mutant and 1p/19q-codeleted	0.97	0.95
	Glioblastoma, IDH-wildtype	0.84	0.87
Sensitivity	Astrocytoma, IDH-mutant	0.60	0.80
	Oligodendroglioma, IDH-mutant and 1p/19q-codeleted	1.00	1.00
	Glioblastoma, IDH-wildtype	0.84	0.84
Specificity	Astrocytoma, IDH-mutant	0.85	0.88
	Oligodendroglioma, IDH-mutant and 1p/19q-codeleted	0.97	0.95
	Glioblastoma, IDH-wildtype	0.83	1.00

Positive predictive value	Astrocytoma, IDH-mutant	0.38	0.50
	Oligodendroglioma, IDH-mutant and 1p/19q-codeleted	0.50	0.33
	Glioblastoma, IDH-wildtype	0.96	1.00
Negative predictive value	Astrocytoma, IDH-mutant	0.93	0.97
	Oligodendroglioma, IDH-mutant and 1p/19q-codeleted	1.00	1.00
	Glioblastoma, IDH-wildtype	0.50	0.55

Caption: Table S3 shows the diagnostic prediction performance, with and without GBCA-enhanced scans, per rater in the development dataset (n=38).
Note: *Evaluations with GBCA-free MRI were based on pre-contrast T1-weighted, T2-weighted, FLAIR, DWI/ADC, and* SWI (if present) *sequences. Evaluations with GBCA-enhanced MRI included post-contrast T1-weighted images in addition to GBCA-free MRI sequences.*
Abbreviations: IDH = isocitrate dehydrogenase, GBCA = gadolinium-based contrast agent

Table S4. Prediction performance of the raters in the optimization dataset			
**Results**		**Rater 1**	**Rater 2**
**Histopathological grade with GBCA-free MRI**			
Accuracy		0.97	0.90
Sensitivity		1.00	0.91
Specificity		0.89	0.89
Positive predictive value		0.96	0.95
Negative predictive value		1.00	0.80
**Histopathological grade with GBCA-enhanced MRI**			
Accuracy		0.87	0.97
Sensitivity		0.67	1.00
Specificity		0.95	0.89
Positive predictive value		0.86	0.96
Negative predictive value		0.88	1.00
**Molecular diagnosis with GBCA-free MRI**			
Overall accuracy *(astrocytoma, IDH-mutant vs. oligodendroglioma, IDH-mutant and 1p/19q-codeleted vs. glioblastoma, IDH-wildtype)*		0.81	0.84
Accuracy	Astrocytoma, IDH-mutant	0.84	0.94
	Oligodendroglioma, IDH-mutant and 1p/19q-codeleted	0.94	0.90
	Glioblastoma, IDH-wildtype	0.84	0.84
Sensitivity	Astrocytoma, IDH-mutant	0.84	0.78
	Oligodendroglioma, IDH-mutant and 1p/19q-codeleted	0.94	0.71
	Glioblastoma, IDH-wildtype	0.84	0.93
Specificity	Astrocytoma, IDH-mutant	0.67	1.00
	Oligodendroglioma, IDH-mutant and 1p/19q-codeleted	0.71	0.96
	Glioblastoma, IDH-wildtype	0.93	0.75
Positive predictive value	Astrocytoma, IDH-mutant	0.75	1.00
	Oligodendroglioma, IDH-mutant and 1p/19q-codeleted	1.00	0.83
	Glioblastoma, IDH-wildtype	0.78	0.78
Negative predictive value	Astrocytoma, IDH-mutant	0.87	0.92
	Oligodendroglioma, IDH-mutant and 1p/19q-codeleted	0.92	0.92
	Glioblastoma, IDH-wildtype	0.92	0.92
**Molecular diagnosis with GBCA-enhanced MRI**			
Overall accuracy *(astrocytoma, IDH-mutant vs. oligodendroglioma, IDH-mutant and 1p/19q-codeleted vs. glioblastoma, IDH-wildtype)*		0.90	0.87
Accuracy	Astrocytoma, IDH-mutant	0.90	0.94
	Oligodendroglioma, IDH-mutant and 1p/19q-codeleted	0.94	0.90
	Glioblastoma, IDH-wildtype	0.97	0.90
Sensitivity	Astrocytoma, IDH-mutant	0.90	0.88
	Oligodendroglioma, IDH-mutant and 1p/19q-codeleted	0.94	0.71
	Glioblastoma, IDH-wildtype	0.97	0.93
Specificity	Astrocytoma, IDH-mutant	0.89	0.95
	Oligodendroglioma, IDH-mutant and 1p/19q-codeleted	0.86	0.96
	Glioblastoma, IDH-wildtype	0.93	0.88
	Astrocytoma, IDH-mutant	0.80	0.89

Positive predictive value	Oligodendroglioma, IDH-mutant and 1p/19q-codeleted	0.86	0.83
	Glioblastoma, IDH-wildtype	1.00	0.88
Negative predictive value	Astrocytoma, IDH-mutant	0.95	0.95
	Oligodendroglioma, IDH-mutant and 1p/19q-codeleted	0.96	0.92
	Glioblastoma, IDH-wildtype	0.94	0.93

Caption: Table S4 shows the diagnostic prediction performance, with and without GBCA-enhanced scans, per rater in the optimization dataset (n=31).
Note: *Evaluations with GBCA-free MRI were based on pre-contrast T1-weighted, T2-weighted, FLAIR, DWI/ADC, and* SWI (if present) *sequences using the Diagnosis Prediction Decision Tree (DPDT). Evaluations with GBCA-enhanced MRI included post-contrast T1-weighted images in addition to the assessment conducted with GBCA-free MRI.*
Abbreviations: IDH = isocitrate dehydrogenase, GBCA = gadolinium-based contrast agent

Table S5. Failed GBCA-free and GBCE-enhanced diagnosis predictions across various tumor locations

Tumor locations	Failed GBCA-free predictions (%)		Failed GBCA-enhanced predictions (%)	
	Tumor grade	Molecular diagnosis	Tumor grade	Molecular diagnosis
Frontal	21%	32%	20%	31%
Parietal	4%	15%	3%	15%
Insula	19%	17%	19%	17%
Temporal	8%	21%	9%	19%
Thalamus	25%	46%	25%	42%
Occipital	15%	19%	10%	17%
Corpus callosum	0%	17%	0%	17%

Caption: Table S5 shows failed diagnosis predictions, including tumor grade and molecular diagnosis, across different tumor locations using GBCA-free and GBCA-enhanced scans based on a combined dataset of all raters.
Abbreviations: GBCA= gadolinium-based contrast agent, GBCA-free scans = pre-contrast T1-weighted, T2-weighted, FLAIR, DWI/ADC, SWI (if present) sequences, GBCA-enhanced scans = pre-contrast T1-weighted, T2-weighted, FLAIR, DWI/ADC, SWI (if present) + post-contrast T1-weighted sequences
Note: Tumor grade prediction includes low-grade (grade 2) vs. high-grade (grade 3/4) gliomas. Molecular diagnosis prediction includes astrocytoma, IDH-mutant vs. oligodendroglioma, IDH-mutant, and 1p/19q-codeleted vs. glioblastoma, IDH-wildtype.

**Table S6. Pairwise inter-rater agreement in the prediction of the histomolecular diagnosis**			
**Histomolecular diagnosis prediction**	**Raters 1&2**	**Raters 1&3**	**Raters 2&3**
Tumor grade $_{\text{GBCA-free}}$* *PABAK*	0.53 (95%-CI: 0.40-0.66) *0.74*	0.52 (95%-CI: 0.38-0.65) *0.74*	0.65 (95%-CI: 0.52-0.78) *0.84*
Tumor grade $_{\text{GBCA-enhanced}}$* *PABAK*	0.74 (95%-CI: 0.63-0.86) *0.87*	0.69 (95%-CI: 0.57-0.81) *0.86*	0.59 (95%-CI: 0.46-0.73) *0.82*
Molecular diagnosis $_{\text{GBCA-free}}$*	0.75 (95%-CI: 0.68-0.82)	0.73 (95%-CI: 0.65-0.80)	0.77 (95%-CI: 0.70-0.84)
Molecular diagnosis $_{\text{GBCA-enhanced}}$*	0.78 (95%-CI: 0.71-0.85)	0.74 (95%-CI: 0.66-0.81)	0.79 (95%-CI: 0.72-0.86)

Caption: Table S6 shows the pairwise inter-rater agreement results for the prediction of histomolecular diagnosis using GBCA-free and GBCA-enhanced scans.
*unweighted Cohen`s kappa
Abbreviations: GBCA= gadolinium-based contrast agent, GBCA-free scans = pre-contrast T1-weighted, T2-weighted, FLAIR, DWI/ADC, SWI (if present) sequences, GBCA-enhanced scans = pre-contrast T1-weighted, T2-weighted, FLAIR, DWI/ADC, SWI (if present) + post-contrast T1-weighted sequences, PABAK = Prevalence-adjusted and bias-adjusted kappa
Note: *Tumor grade prediction includes low-grade (grade 2) vs. high-grade (grade 3/4) gliomas. Molecular diagnosis prediction includes astrocytoma, IDH-mutant vs. oligodendroglioma, IDH-mutant and 1p/19q-codeleted vs. glioblastoma, IDH-wildtype. Interpretation of agreement values was as follows: 0.01–0.20, slight; 0.21–0.40, fair; 0.41–0.60, moderate; 0.61–0.80, substantial; and 0.81–0.99, almost perfect.*

Table S7. Pairwise inter-rater agreement in the evaluation of imaging features included in DPDT

Imaging features	Raters 1&2	Raters 1&3	Raters 2&3
Necrosis $_{GBCA-free}$* *PABAK*	0.87 (95%-CI: 0.81-0.93) *0.88*	0.80 (95%-CI: 0.73-0.87) *0.82*	0.82 (95%-CI: 0.75-0.88) *0.83*
Necrosis $_{GBCA-enhanced}$* *PABAK*	0.85 (95%-CI: 0.79-0.92) *0.87*	0.84 (95%-CI: 0.77-0.91) *0.86*	0.86 (95%-CI: 0.80-0.92) *0.87*
Diffusion restriction $_{GBCA-free}$**	0.42 (95%-CI: 0.34-0.49)	0.39 (95%-CI: 0.31-0.47)	0.37 (95%-CI: 0.28-0.46)
Diffusion restriction $_{GBCA-enhanced}$**	0.42 (95%-CI: 0.34-0.50)	0.34 (95%-CI: 0.26-0.42)	0.43 (95%-CI: 0.33-0.52)
Hemorrhage $_{GBCA-free}$* *PABAK*	0.65 (95%-CI: 0.55-0.74) *0.72*	0.48 (95%-CI: 0.35-0.61) *0.73*	0.33 (95%-CI: 0.22-0.43) *0.55*
Hemorrhage $_{GBCA-enhanced}$* *PABAK*	0.54 (95%-CI: 0.44-0.64) *0.64*	0.40 (95%-CI: 0.26-0.53) *0.70*	0.29 (95%-CI: 0.19-0.39) *0.50*
T2-FLAIR mismatch sign $_{GBCA-free}$* *PABAK*	0.52 (95%-CI: 0.27-0.78) *0.93*	0.60 (95%-CI: 0.31-0.89) *0.95*	0.59 (95%-CI: 0.36-0.82) *0.93*
T2-FLAIR mismatch sign $_{GBCA-enhanced}$* *PABAK*	0.53 (95%-CI: 0.27-0.78) *0.93*	0.51 (95%-CI: 0.23-0.80) *0.93*	0.60 (95%-CI: 0.45-0.88) *0.94*
Nonenhancing tumor margin $_{GBCA-free}$* *PABAK*	0.35 (95%-CI: 0.20-0.50) *0.74*	0.36 (95%-CI: 0.22-0.51) *0.71*	0.58 (95%-CI: 0.45-0.70) *0.78*
Nonenhancing tumor margin $_{GBCA-enhanced}$* *PABAK*	0.43 (95%-CI: 0.28-0.57) *0.74*	0.47 (95%-CI: 0.34-0.61) *0.73*	0.49 (95%-CI: 0.37-0.62) *0.71*
T2 homogeneity $_{GBCA-free}$* *PABAK*	0.44 (95%-CI: 0.28-0.60) *0.80*	0.39 (95%-CI: 0.22-0.55) *0.77*	0.49 (95%-CI: 0.28-0.69) *0.87*
T2 homogeneity $_{GBCA-enhanced}$* *PABAK*	0.42 (95%-CI: 0.23-0.60) *0.82*	0.40 (95%-CI: 0.22-0.58) *0.81*	0.57 (95%-CI: 0.39-0.75) *0.88*
Calvarial remodeling $_{GBCA-free}$* *PABAK*	0.54 (95%-CI: 0.35-0.74) *0.89*	0.35 (95%-CI: 0.13-0.58) *0.87*	0.56 (95%-CI: 0.36-0.76) *0.90*
Calvarial remodeling $_{GBCA-enhanced}$* *PABAK*	0.58 (95%-CI: 0.40-0.77) *0.89*	0.53 (95%-CI: 0.31-0.75) *0.91*	0.56 (95%-CI: 0.35-0.76) *0.91*
Cyst $_{GBCA-free}$* *PABAK*	0.42 (95%-CI: 0.23-0.62) *0.85*	0.36 (95%-CI: 0.17-0.55) *0.84*	0.46 (95%-CI: 0.19-0.72) *0.91*
Cyst $_{GBCA-enhanced}$* *PABAK*	0.47 (95%-CI: 0.27-0.68) *0.87*	0.13 (95%-CI: -0.04-0.31) *0.82*	0.45 (95%-CI: 0.19-0.71) *0.91*
Calcification $_{GBCA-free}$* *PABAK*	0.73 (95%-CI: 0.45-1.02) *0.90*	0.55 (95%-CI: 0.21-0.88) *0.84*	0.54 (95%-CI: 0.13-0.95) *0.87*
Calcification $_{GGBCA-enhanced}$* *PABAK*	0.80 (95%-CI: 0.52-1.07) *0.94*	0.49 (95%-CI: 0.08-0.89) *0.84*	0.62 (95%-CI: 0.21-1.04) *0.90*
Midline shift $_{GBCA-free}$* *PABAK*	0.64 (95%-CI: 0.56-0.73) *0.68*	0.60 (95%-CI: 0.51-0.69) *0.62*	0.80 (95%-CI: 0.73-0.86) *0.80*
Midline shift $_{GBCA-enhanced}$* *PABAK*	0.73 (95%-CI: 0.65-0.82) *0.76*	0.69 (95%-CI: 0.61-0.77) *0.71*	0.78 (95%-CI: 0.71-0.85) *0.79*
Substantial edema $_{GBCA-free}$* *PABAK*	0.53 (95%-CI: 0.43-0.64) *0.66*	0.51 (95%-CI: 0.42-0.60) *0.50*	0.25 (95%-CI: 0.18-0.32) *0.21*
Substantial edema $_{GBCA-enhanced}$* *PABAK*	0.45 (95%-CI: 0.34-0.57) *0.62*	0.50 (95%-CI: 0.41-0.59) *0.50*	0.28 (95%-CI: 0.20-0.35) *0.27*

Caption: Table S7 shows the pairwise inter-rater agreement results for imaging features involved in DPDT using both GBCA-free and GBCA-enhanced scans.
*unweighted Cohen`s kappa, **weighted Cohen's kappa
Abbreviations: DPDT = diagnosis prediction decision tree, GBCA= gadolinium-based contrast agent, GBCA-free scans = pre-contrast T1-weighted, T2-weighted, FLAIR, DWI/ADC, SWI (if present) sequences, GBCA-enhanced

scans = pre-contrast T1-weighted, T2-weighted, FLAIR, DWI/ADC, SWI (if present) + post-contrast T1-weighted sequences, PABAK = Prevalence-adjusted and bias-adjusted kappa
Note: *The interpretation of agreement values was as follows: 0.01–0.20, slight; 0.21–0.40, fair; 0.41–0.60, moderate; 0.61–0.80, substantial; and 0.81–0.99, almost perfect.*

Supplementary Figures and Figure Captions

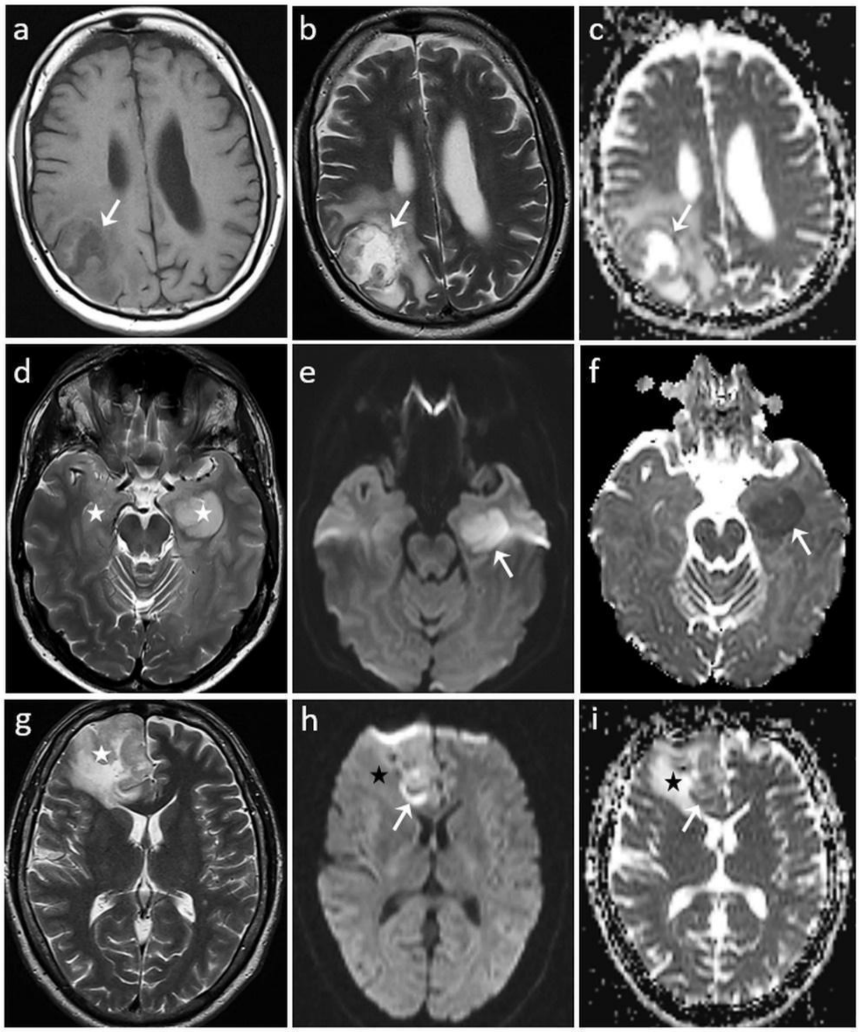

Figure S1. Case examples for the evaluation of necrosis and diffusion.
Necrosis (a,b,c; glioblastoma, IDH-wildtype): Axial MR images show a right parietal lesion with typical features of central necrosis characterized by T1 hypointense (a), T2 hyperintense (b) and high fluid-like ADC signal (c) covered by irregular and thick margins (white arrows). ***Restricted diffusion (d,e,f; glioblastoma, IDH-wildtype):*** Axial MR images show left and right mesial temporal/hippocampal infiltrative T2 hyperintense lesions (d, white stars) with restricted diffusion on the left side characterized by high TRACE/DWI signal (e; white arrow) and corresponding low ADC signal (f, white arrow). ***Dubious and facilitated diffusion (g,h,i; high-grade oligodendroglioma, IDH-mutant and 1p/19q-codeleted):*** Axial MR images demonstrate right frontal T2 hyperintense lesion (g, white star) with dubious diffusion characterized by high TRACE/DWI signal (h, white arrow) plus corresponding intermediate healthy cortex-like ADC signal (i, white arrow), and facilitated diffusion characterized by low TRACE/DWI signal (h, black star) plus corresponding high ADC signal (i, black star).

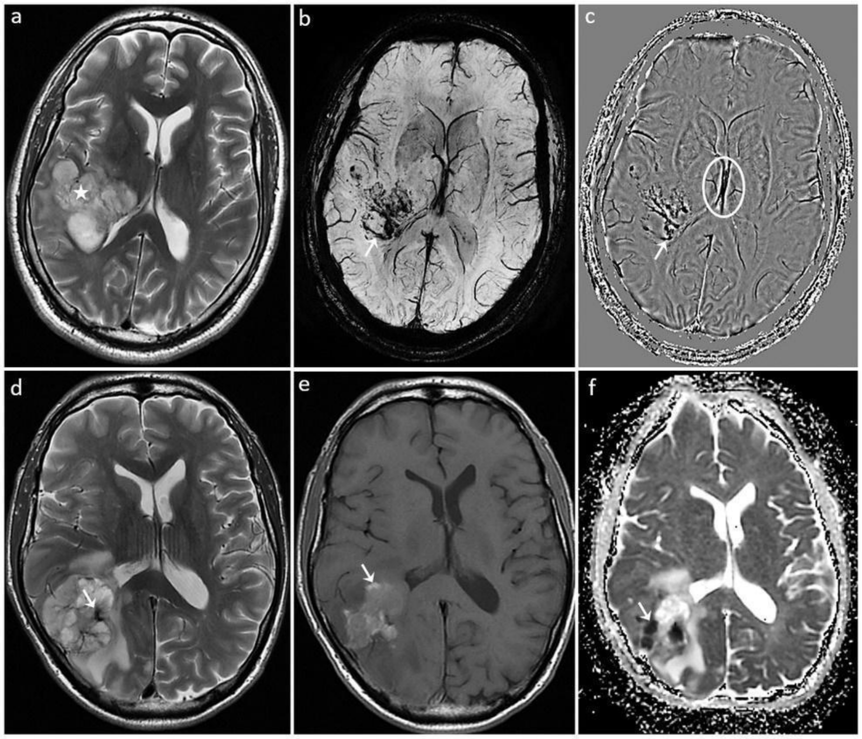

Figure S2. Case examples for the evaluation of hemorrhage.

Hemorrhage on SWI (a,b,c; glioblastoma, IDH-wildtype): Axial MR images show a right temporal T2 hyperintense lesion (a, white star) with hemorrhage characterized by the blooming on post-processed SWI image (b, white arrow) with the same signal intensity as internal cerebral veins on filtered phase images (c, white arrow pointing out hemorrhage and white circle highlighting internal cerebral vein). ***Hemorrhage on pre-contrast T1-weighted image (d,e,f; glioblastoma, IDH-wildtype):*** Axial MR images demonstrate right temporoparietal lesion with hemorrhagic necrosis characterized by a hyperintense signal within the necrotic part of the tumor on pre-contrast T1-weighted image (e, white arrow). There are also T2 hypointensity (d, white arrow) and low ADC signal (f, white arrow) related to hemorrhage. SWI images were not acquired for this case.

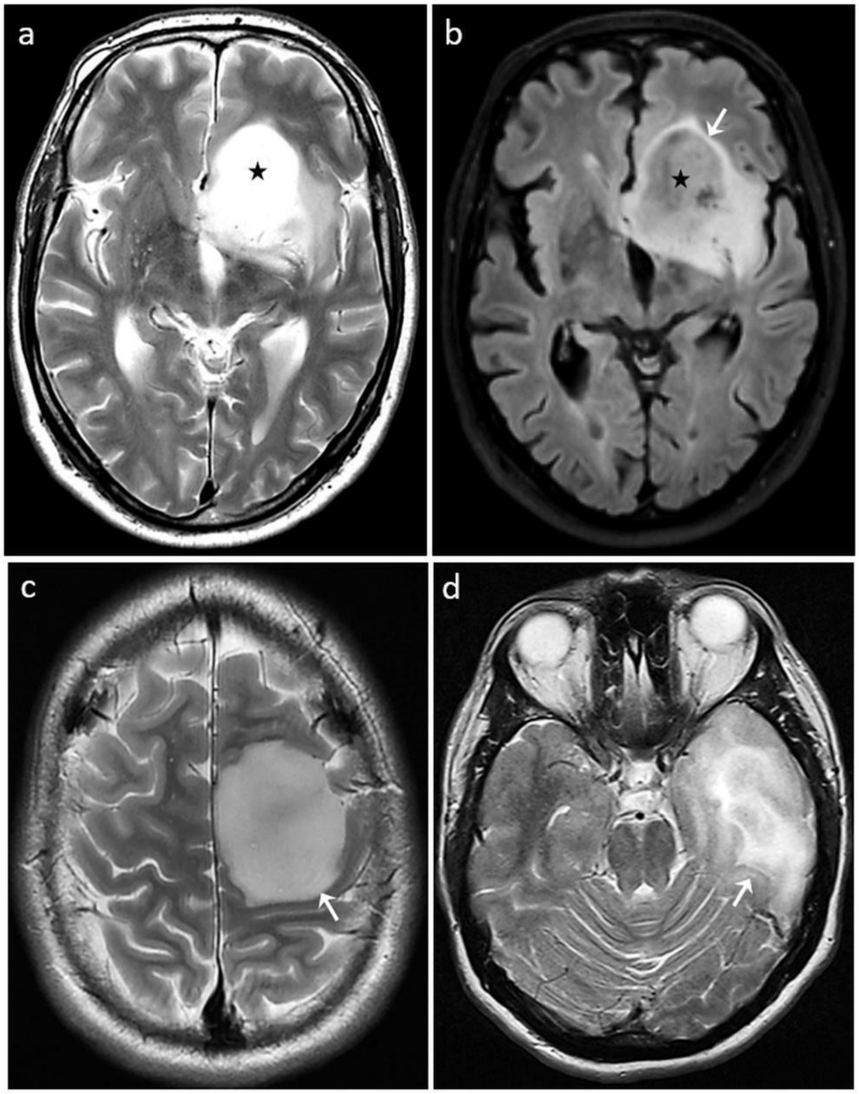

Figure S3. Case examples for the evaluation of T2-FLAIR mismatch sign, non-enhancing tumor margin, and T2 signal homogeneity.

T2-FLAIR mismatch sign (a,b; low-grade astrocytoma, IDH-mutant): Axial T2 weighted image shows left fronto-insular hyperintense nearly-homogeneous hyperintense lesion (a, black star) and FLAIR image demonstrates near-complete signal drop-out in the lesion (b, black star) except for a hyperintense peripheral rim (b, white arrow). ***Well-defined margin and T2 homogeneity (c; low-grade astrocytoma, IDH-mutant):*** Axial T2 weighted image shows left frontal lesion with homogeneous hyperintense signal and well-defined margins (white arrow). ***Ill-defined margin and T2 inhomogeneity (d; high-grade astrocytoma, IDH-mutant):*** Axial T2 weighted image shows left temporal lesion with inhomogeneous hyperintense signal and ill-defined margins (white arrow).

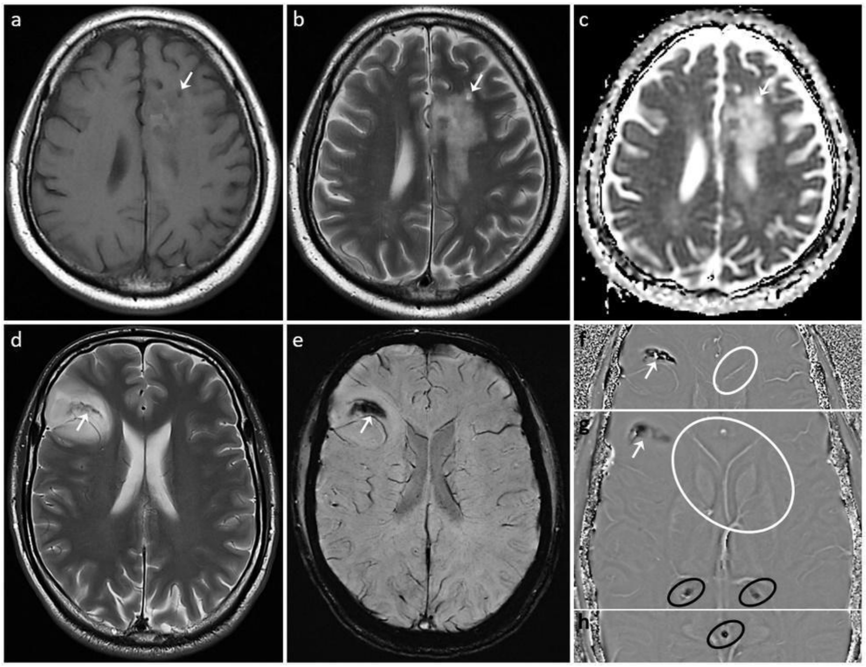

Figure S4. Case examples for the evaluation of cyst and calcification.

Cyst (a,b,c; high-grade oligodendroglioma, IDH-mutant and 1p/19q-codeleted): Axial MR images show a left frontal lesion consisting of eccentrically located small cyst with regular and smooth margins characterized by a well-defined rounded area of low T1-weighted (a, white arrow), bright T2-weighted (b, white arrow) and high ADC signal (c, white arrow) matching cerebrospinal fluid signal intensity. ***Calcification (d,e,f,g,h; low-grade oligodendroglioma, IDH-mutant and 1p/19q-codeleted):*** Axial MR images show right frontal T2 hyperintense heterogeneous lesion with internal calcification (d, white arrow) characterized by the blooming on post-processed SWI image (e, white arrow). This area displays a mainly hypointense signal on filtered phase image (f (the same level as in image e) and g (the choroid plexus level, white arrows), similar to calcified choroid plexus (g, dark circles) and pineal gland (h, dark circle), and opposite to the internal cerebral veins (f and g, white circles). These signal features suggest calcification rather than hemorrhage, using the internal cerebral veins as a reference for hemorrhage and the calcified choroid plexus/pineal gland as a reference for calcification.

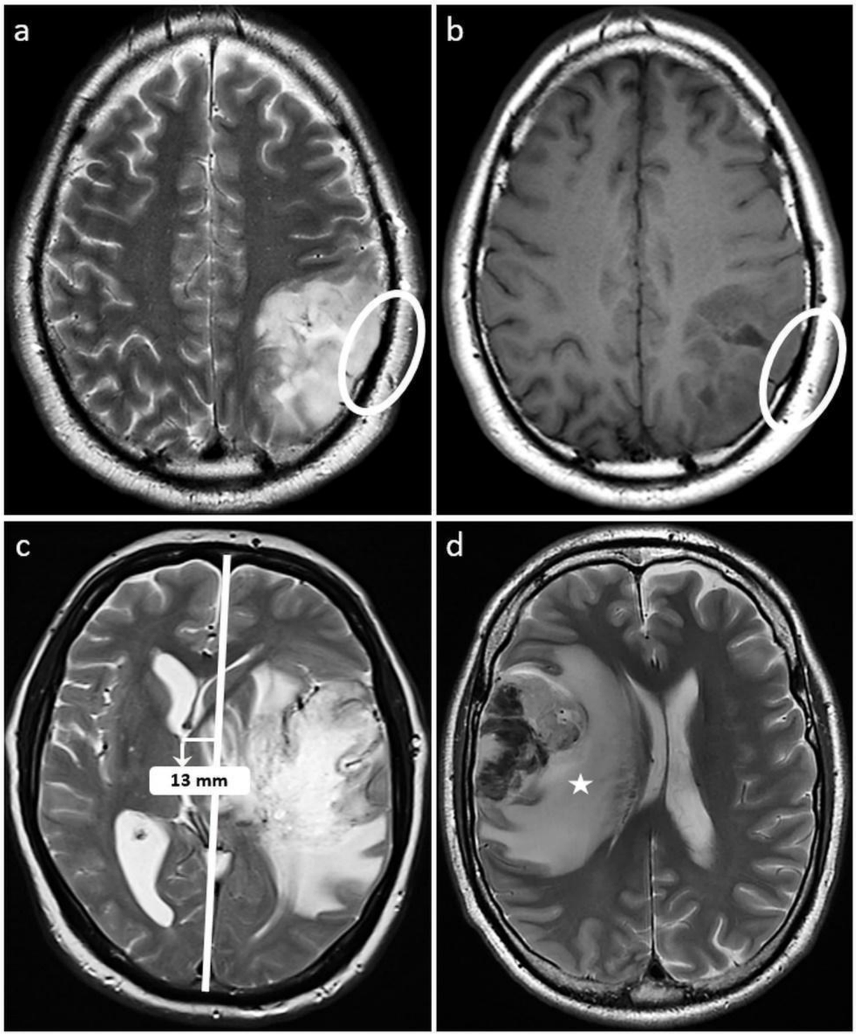

Figure S5. Case examples for the evaluation of calvarial remodeling, midline shift, and substantial edema.

Calvarial remodeling (a,b; low-grade oligodendroglioma, IDH-mutant and 1p/19q-codeleted): Axial T2-weighted (a) and pre-contrast T1-weighted (b) images show left parietal heterogeneous lesion causing discernible thinning/ remodeling of the inner table of parietal bone (white circles). ***Midline shift (c; glioblastoma, IDH-wildtype):*** Axial T2 weighted image shows left temporo-insular heterogeneous lesion causing more than 5 mm shift of the midline structures (horizontal line and text box indicating the midline shift degree of 13 mm) to the right according to the line drawn coplanar with falx connecting the anterior and posterior superior sagittal sinus attachments (white vertical line). ***Substantial edema (d; glioblastoma, IDH-wildtype):*** Axial T2-weighted image shows a right frontal heterogeneous lesion with hemorrhagic central necrosis lesion causing edema ≥50% of the tumor volume (white star).

Development dataset

Rater 1

True: reference standard	Predicted GBCA-free grade	
	Low-grade glioma	High-grade glioma
Low-grade glioma	4	1
High-grade glioma	1	32

True: reference standard	Predicted GBCA-enhanced grade	
	Low-grade glioma	High-grade glioma
Low-grade glioma	4	1
High-grade glioma	0	33

True: reference standard	Predicted GBCA-free molecular diagnosis		
	Oligo, IDH-mut & 1p/19q-codel.	Astro, IDH-mut	GB, IDH-wt
Oligo, IDH-mut & 1p/19q-codel.	1	0	0
Astro, IDH-mut	2	3	0
GB, IDH-wt	0	7	25

True: reference standard	Predicted GBCA-enhanced molecular diagnosis		
	Oligo, IDH-mut & 1p/19q-codel.	Astro, IDH-mut	GB, IDH-wt
Oligo, IDH-mut & 1p/19q-codel.	1	0	0
Astro, IDH-mut	1	3	1
GB, IDH-wt	0	5	27

Rater 2

True: reference standard	Predicted GBCA-free grade	
	Low-grade glioma	High-grade glioma
Low-grade glioma	4	1
High-grade glioma	3	30

True: reference standard	Predicted GBCA-enhanced grade	
	Low-grade glioma	High-grade glioma
Low-grade glioma	4	1
High-grade glioma	4	29

True: reference standard	Predicted GBCA-free molecular diagnosis		
	Oligo, IDH-mut & 1p/19q-codel.	Astro, IDH-mut	GB, IDH-wt
Oligo, IDH-mut & 1p/19q-codel.	1	0	0
Astro, IDH-mut	1	4	0
GB, IDH-wt	3	6	23

True: reference standard	Predicted GBCA-enhanced molecular diagnosis		
	Oligo, IDH-mut & 1p/19q-codel.	Astro, IDH-mut	GB, IDH-wt
Oligo, IDH-mut & 1p/19q-codel.	1	0	0
Astro, IDH-mut	1	4	0
GB, IDH-wt	1	4	27

Figure S6. Per-rater confusion matrices for predicting histomolecular diagnosis of adult-type diffuse gliomas using GBCA-free and GBCA-enhanced scans in the development dataset.

Diagnosis predictions include tumor grade (low-grade glioma: grade 2 vs. high-grade glioma: grade 3/4) and molecular status (astrocytoma, IDH-mutant vs. oligodendroglioma, IDH-mutant and 1p/19q-codeleted vs. glioblastoma, IDH-wildtype) evaluations. The reference standard is the histomolecular diagnosis. Green and red color boxes show correct and false predictions, respectively. *GBCA = Gadolinium-based contrast agent; IDH = isocitrate dehydrogenase;* Oligo, IDH-mut & 1p/19-codel. = oligodendroglioma; IDH-mutant and 1p/19q-codeleted; Astro, IDH-mut= astrocytoma, IDH-mutant; GB, IDH-wt = glioblastoma, IDH-wildtype

Optimization dataset

Rater 1

True: reference standard	Predicted GBCA-free grade	
	Low-grade glioma	High-grade glioma
Low-grade glioma	8	1
High-grade glioma	0	22

True: reference standard	Predicted GBCA-enhanced grade	
	Low-grade glioma	High-grade glioma
Low-grade glioma	6	3
High-grade glioma	1	21

True: reference standard	Predicted GBCA-free molecular diagnosis		
	Oligo, IDH-mut & 1p/19q-codel.	Astro, IDH-mut	GB, IDH-wt
Oligo, IDH-mut & 1p/19q-codel.	5	1	1
Astro, IDH-mut	0	6	3
GB, IDH-wt	0	1	14

True: reference standard	Predicted GBCA-enhanced molecular diagnosis		
	Oligo, IDH-mut & 1p/19q-codel.	Astro, IDH-mut	GB, IDH-wt
Oligo, IDH-mut & 1p/19q-codel.	6	1	0
Astro, IDH-mut	1	8	0
GB, IDH-wt	0	1	14

Rater 2

True: reference standard	Predicted GBCA-free grade	
	Low-grade glioma	High-grade glioma
Low-grade glioma	8	1
High-grade glioma	2	20

True: reference standard	Predicted GBCA-enhanced grade	
	Low-grade glioma	High-grade glioma
Low-grade glioma	8	1
High-grade glioma	0	22

True: reference standard	Predicted GBCA-free molecular diagnosis		
	Oligo, IDH-mut & 1p/19q-codel.	Astro, IDH-mut	GB, IDH-wt
Oligo, IDH-mut & 1p/19q-codel.	5	0	2
Astro, IDH-mut	0	7	2
GB, IDH-wt	1	0	14

True: reference standard	Predicted GBCA-enhanced molecular diagnosis		
	Oligo, IDH-mut & 1p/19q-codel.	Astro, IDH-mut	GB, IDH-wt
Oligo, IDH-mut & 1p/19q-codel.	5	1	1
Astro, IDH-mut	0	8	1
GB, IDH-wt	1	0	14

Figure S7. Per-rater confusion matrices for predicting histomolecular diagnosis of adult-type diffuse gliomas using GBCA-free and GBCA-enhanced scans in the optimization dataset.

Diagnosis predictions include tumor grade (low-grade glioma: grade 2 vs. high-grade glioma: grade 3/4) and molecular status (astrocytoma, IDH-mutant vs. oligodendroglioma, IDH-mutant and 1p/19q-codeleted vs. glioblastoma, IDH-wildtype) evaluations. The reference standard is the histomolecular diagnosis. Green and red color boxes show correct and false predictions, respectively. *GBCA = Gadolinium-based contrast agent; IDH = isocitrate dehydrogenase;* Oligo, IDH-mut & 1p/19-codel. = oligodendroglioma; IDH-mutant and 1p/19q-codeleted; Astro, IDH-mut= astrocytoma, IDH-mutant; GB, IDH-wt = glioblastoma, IDH-wildtype

Test dataset

Rater 1

True: reference standard	Predicted GBCA-free grade	
	Low-grade glioma	High-grade glioma
Low-grade glioma	32	22
High-grade glioma	25	224

True: reference standard	Predicted GBCA-enhanced grade	
	Low-grade glioma	High-grade glioma
Low-grade glioma	30	24
High-grade glioma	13	236

True: reference standard	Predicted GBCA-free molecular diagnosis		
	Oligo, IDH-mut & 1p/19q-codel.	Astro, IDH-mut	GB, IDH-wt
Oligo, IDH-mut & 1p/19q-codel.	14	12	8
Astro, IDH-mut	12	26	10
GB, IDH-wt	2	25	194

True: reference standard	Predicted GBCA-enhanced molecular diagnosis		
	Oligo, IDH-mut & 1p/19q-codel.	Astro, IDH-mut	GB, IDH-wt
Oligo, IDH-mut & 1p/19q-codel.	12	14	8
Astro, IDH-mut	9	24	15
GB, IDH-wt	3	22	196

Rater 2

True: reference standard	Predicted GBCA-free grade	
	Low-grade glioma	High-grade glioma
Low-grade glioma	30	24
High-grade glioma	12	237

True: reference standard	Predicted GBCA-enhanced grade	
	Low-grade glioma	High-grade glioma
Low-grade glioma	29	25
High-grade glioma	15	234

True: reference standard	Predicted GBCA-free molecular diagnosis		
	Oligo, IDH-mut & 1p/19q-codel.	Astro, IDH-mut	GB, IDH-wt
Oligo, IDH-mut & 1p/19q-codel.	12	15	7
Astro, IDH-mut	11	28	9
GB, IDH-wt	4	27	190

True: reference standard	Predicted GBCA-enhanced molecular diagnosis		
	Oligo, IDH-mut & 1p/19q-codel.	Astro, IDH-mut	GB, IDH-wt
Oligo, IDH-mut & 1p/19q-codel.	13	16	5
Astro, IDH-mut	9	30	9
GB, IDH-wt	5	26	190

Rater 3

True: reference standard	Predicted GBCA-free grade	
	Low-grade glioma	High-grade glioma
Low-grade glioma	25	29
High-grade glioma	13	236

True: reference standard	Predicted GBCA-enhanced grade	
	Low-grade glioma	High-grade glioma
Low-grade glioma	25	29
High-grade glioma	11	238

True: reference standard	Predicted GBCA-free molecular diagnosis		
	Oligo, IDH-mut & 1p/19q-codel.	Astro, IDH-mut	GB, IDH-wt
Oligo, IDH-mut & 1p/19q-codel.	10	19	5
Astro, IDH-mut	7	30	11
GB, IDH-wt	2	32	187

True: reference standard	Predicted GBCA-enhanced molecular diagnosis		
	Oligo, IDH-mut & 1p/19q-codel.	Astro, IDH-mut	GB, IDH-wt
Oligo, IDH-mut & 1p/19q-codel.	9	21	4
Astro, IDH-mut	6	32	10
GB, IDH-wt	1	26	194

Figure S8. Per-rater confusion matrices for predicting histomolecular diagnosis of adult-type diffuse gliomas using GBCA-free and GBCA-enhanced scans in the test dataset. Diagnosis predictions include tumor grade (low-grade glioma: grade 2 vs. high-grade glioma: grade 3/4) and molecular status (astrocytoma, IDH-mutant vs. oligodendroglioma, IDH-mutant and 1p/19q-codeleted vs. glioblastoma, IDH-wildtype) evaluations. The reference standard is the histomolecular diagnosis. Green and red color boxes show correct and false predictions, respectively. *GBCA = Gadolinium-based contrast agent; IDH = isocitrate dehydrogenase;* Oligo, IDH-mut & 1p/19-codel. = oligodendroglioma; IDH-mutant and 1p/19q-codeleted; Astro, IDH-mut= astrocytoma, IDH-mutant; GB, IDH-wt = glioblastoma, IDH-wildtype

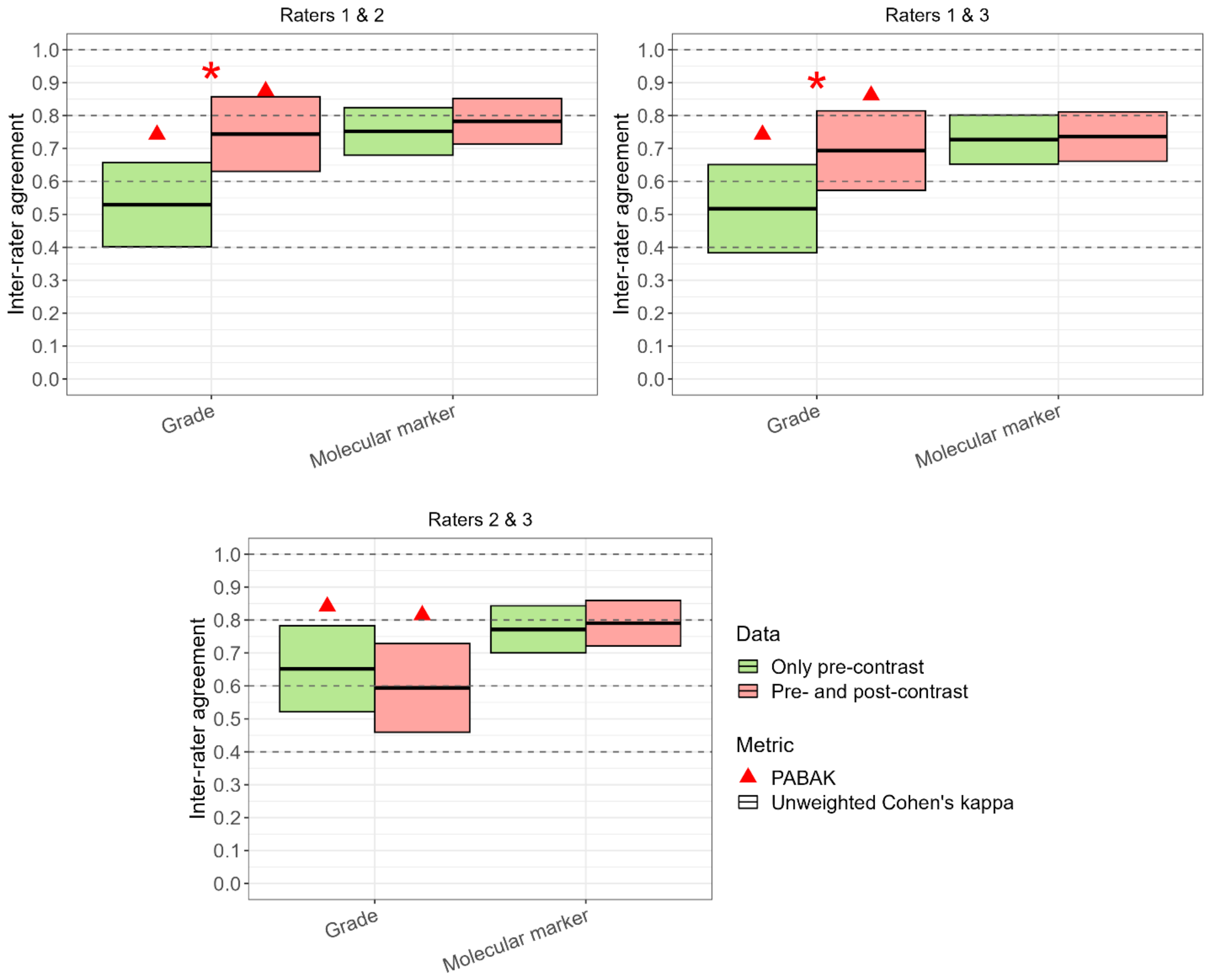

Figure S9. Pairwise inter-rater agreement in histomolecular diagnosis prediction of adult-type diffuse gliomas using GBCA-free vs. GBCA-enhanced scans.

Color box plots show inter-rater agreement in predicting tumor grade (low-grade: grade 2 vs. high-grade: grade 3/4) and molecular status (astrocytoma, IDH-mutant vs. oligodendroglioma, IDH-mutant and 1p/19q-codeleted vs. glioblastoma, IDH-wildtype) among various two raters (rater 1&2, rater 1&3, rater 2&3). Green bars depict the results based on the evaluation of GBCA-free scans (only pre-contrast sequences: pre-contrast T1-weighted, T2-weighted, FLAIR, DWI/ADC, and SWI (if present)) and red bars show the results of the evaluation using GBCA-enhanced scans (pre- and post-contrast sequences: pre-contrast T1-weighted, T2-weighted, FLAIR, DWI/ADC, SWI (if present) + post-contrast T1-weighted). Red triangles indicate prevalence-adjusted and bias-adjusted kappa (PABAK) values that compensate for the possible influence of dataset diagnosis imbalances. Comparison of agreements between GBCA-free and GBCA-enhanced predictions was insignificant ($p > 0.05$) except for agreements in grade prediction for raters 1&2 and raters 1&3 (red stars, $p = 0.003$ and $p = 0.02$, respectively). *Note: The interpretation of agreement values was as follows: 0.01–0.20, slight; 0.21–0.40, fair; 0.41–0.60, moderate; 0.61–0.80, substantial; and 0.81–0.99, almost perfect. GBCA = Gadolinium-based contrast agent, IDH = isocitrate dehydrogenase.*

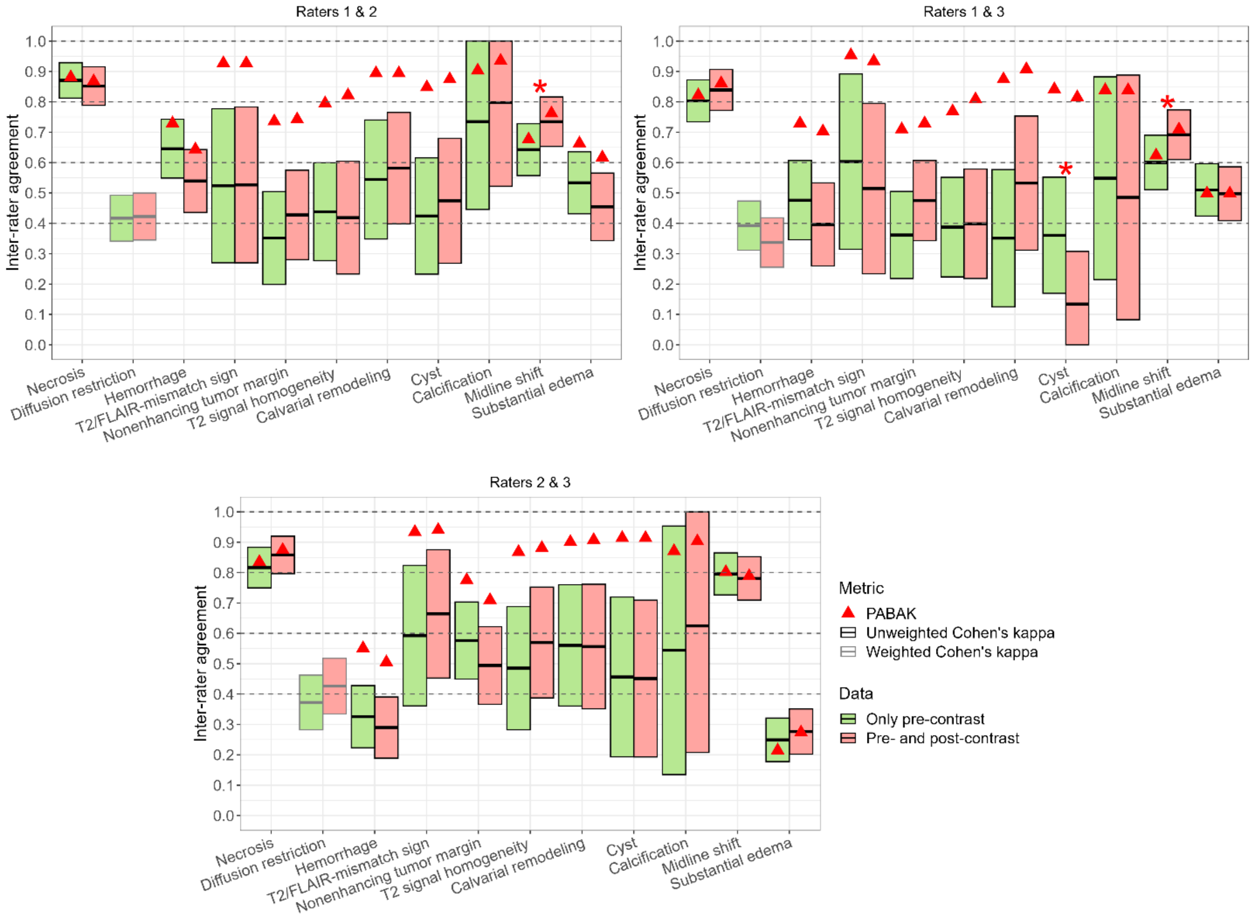

Figure S10. Pairwise inter-rater agreement in the evaluation of imaging features included in the Diagnosis Prediction Decision tree (DPDT) for adult-type diffuse gliomas using GBCA-free vs. GBCA-enhanced scans.

Color box plots show inter-rater agreement in the evaluation of single DPDT imaging features using either GBCA-free or GBCA-enhanced scans among various two raters (rater 1&2, rater 1&3, rater 2&3). Green bars depict the results based on the evaluation of GBCA-free scans (only pre-contrast sequences: pre-contrast T1-weighted, T2-weighted, FLAIR, DWI/ADC and SWI (if present)) and red bars show the results of the evaluation using GBCA-enhanced scans (pre- and post-contrast sequences: pre-contrast T1-weighted, T2-weighted, FLAIR, DWI/ADC, SWI (if present) + post-contrast T1-weighted). Red triangles indicate prevalence-adjusted and bias-adjusted kappa (PABAK) values that compensate for the possible influence of dataset diagnosis imbalances. Comparison of agreements between GBCA-free and GBCA-enhanced assessments was insignificant ($p > 0.05$) except for rater 1&2 midline shift (red star, $p = 0.02$) and rater 1&3 cyst (red star, $p = 0.04$) and midline shift (red star, $p = 0.03$). *Note: The interpretation of agreement values was as follows: 0.01–0.20, slight; 0.21–0.40, fair; 0.41–0.60, moderate; 0.61–0.80, substantial; and 0.81–0.99, almost perfect. GBCA = Gadolinium-based contrast agent.*

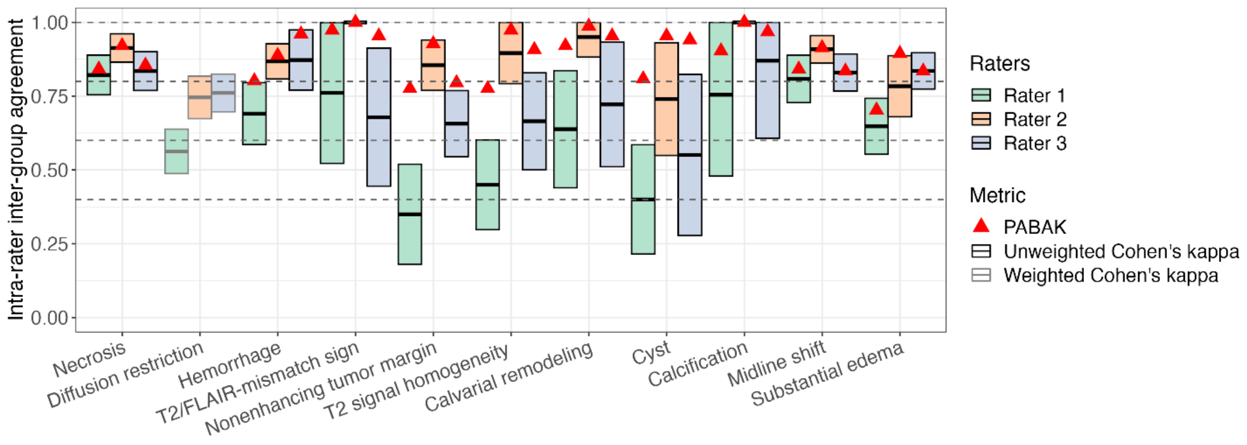

Figure S11. Intra-rater inter-group agreement in the evaluation of imaging features included in the Diagnosis Prediction Decision tree (DPDT) for adult-type diffuse gliomas. Color box plot describes intra-rater inter-group agreement comparing GBCA-free and GBCA-enhanced evaluations of single DPDT imaging features by each rater (rater 1 = green bar, rater 2 = orange bar, rater 3 = blue bar). Red triangles indicate prevalence-adjusted and bias-adjusted kappa (PABAK) values that compensate for the possible influence of dataset diagnosis imbalances. Comparison of agreements among all raters revealed significant differences ($p < 0.05$) except for T2-FLAIR mismatch sign and calcification ($p > 0.05$). *Note: The interpretation of agreement values was as follows: 0.01–0.20, slight; 0.21–0.40, fair; 0.41–0.60, moderate; 0.61–0.80, substantial; and 0.81–0.99, almost perfect. GBCA = Gadolinium-based contrast agent.*
